# Isoprenoid biosynthesis in dandelion latex is enhanced by the overexpression of three key enzymes involved in the mevalonate pathway

**DOI:** 10.1186/s12870-017-1036-0

**Published:** 2017-05-22

**Authors:** Katharina M. Pütter, Nicole van Deenen, Kristina Unland, Dirk Prüfer, Christian Schulze Gronover

**Affiliations:** 1 Institute of Plant Biology and Biotechnology, Schlossplatz 8, 48143 Muenster, Germany; 2 Fraunhofer Institute for Molecular Biology and Applied Ecology (IME), Schlossplatz 8, 48143 Muenster, Germany

**Keywords:** ATP citrate lyase, Acetoacetyl-CoA thiolase, Mevalonate pathway, Taraxacum, Natural rubber, Latex, Triterpenes, Sterols

## Abstract

**Background:**

Latex from the dandelion species *Taraxacum brevicorniculatum* contains many high-value isoprenoid end products, e.g. triterpenes and polyisoprenes such as natural rubber. The isopentenyl pyrophosphate units required as precursors for these isoprenoids are provided by the mevalonate (MVA) pathway. The key enzyme in this pathway is 3-hydroxy-methyl-glutaryl-CoA reductase (HMGR) and its activity has been thoroughly characterized in many plant species including dandelion. However, two enzymes acting upstream of HMGR have not been characterized in dandelion latex: ATP citrate lyase (ACL), which provides the acetyl-CoA utilized in the MVA pathway, and acetoacetyl-CoA thiolase (AACT), which catalyzes the first step in the pathway to produce acetoacetyl-CoA. Here we isolated *ACL* and *AACT* genes from *T. brevicorniculatum* latex and characterized their expression profiles. We also overexpressed the well-characterized *HMGR*, *ACL* and *AACT* genes from *Arabidopsis thaliana* in *T. brevicorniculatum* to determine their impact on isoprenoid end products in the latex.

**Results:**

The spatial and temporal expression profiles of *T. brevicorniculatum ACL* and *AACT* revealed their pivotal role in the synthesis of precursors necessary for isoprenoid biosynthesis in latex. The overexpression of *A. thaliana ACL* and *AACT* and *HMGR* in *T. brevicorniculatum* latex resulted in the accumulation of all three enzymes, increased the corresponding enzymatic activities and ultimately increased sterol levels by ~5-fold and pentacyclic triterpene and *cis*-1,4-isoprene levels by ~2-fold. Remarkably high levels of the triterpene precursor squalene were also detected in the triple-transgenic lines (up to 32 mg/g root dry weight) leading to the formation of numerous lipid droplets which were observed in root cross-sections.

**Conclusions:**

We could show the effective expression of up to three transgenes in *T. brevicorniculatum* latex which led to increased enzymatic activity and resulted in high level squalene accumulation in the dandelion roots up to an industrially relevant amount. Our data provide insight into the regulation of the MVA pathway in dandelion latex and can be used as a basis for metabolic engineering to enhance the production of isoprenoid end products in this specialized tissue.

**Electronic supplementary material:**

The online version of this article (doi:10.1186/s12870-017-1036-0) contains supplementary material, which is available to authorized users.

## Background

**Fig. 1:**
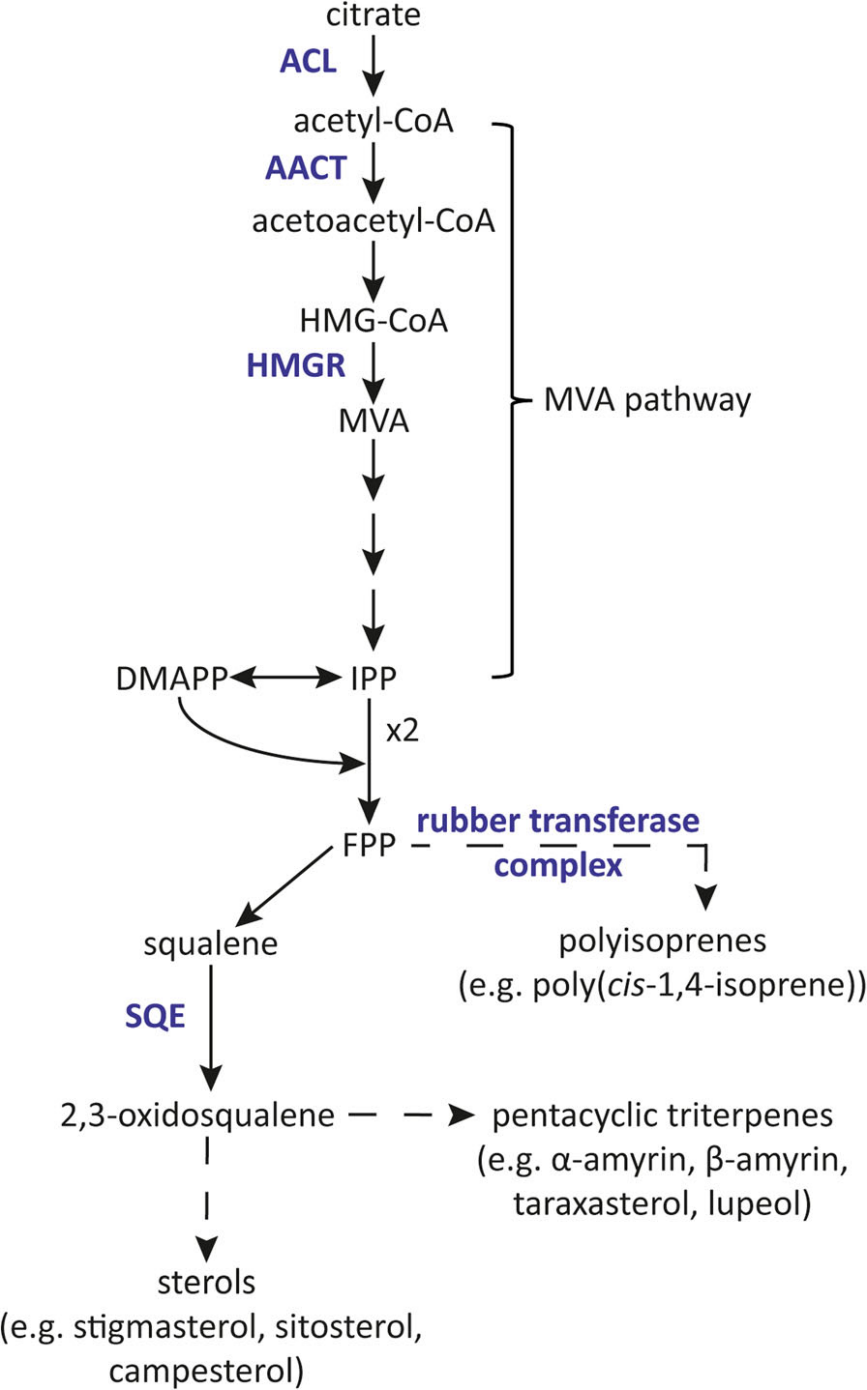
Proposed biosynthesis of isoprenoid products via the MVA pathway with upstream reactions in *T. brevicorniculatum* latex. Enzymes or enzyme complexes are shown in blue and multiple/dashed arrows indicate multiple enzymatic steps. ACL, ATP citrate lyase; AACT, acetoacetyl-CoA thiolase; DMAPP, dimethylallyl pyrophosphate; FPP, farnesyl diphosphate; HMGR, 3-hydroxy-methyl-glutaryl-CoA reductase; IPP, isopentenyl pyrophosphate; MVA, mevalonate; SQE, squalene epoxidase

The dandelion species *Taraxacum brevicorniculatum* belongs to more than 12,500 plant species that produce latex, a milky sap, in specialized parenchyma cells called laticifers [1]. Among other secondary metabolites and proteins, laticifers contain a large variety of industrially valuable isoprenoid end products, including triterpenes such as α-amyrin, β-amyrin, lupeol and taraxasterol, and polyisoprenes such as natural rubber [2]. The components of the rubber transferase complex have been investigated in rubber-producing plants like the rubber tree (*Hevea brasiliensis*), lettuce (*Lactuca sativa*) and *T. brevicorniculatum* [3–5]. Labeling experiments in *H. brasiliensis* showed that the cytosolic mevalonate (MVA) pathway provides the precursor isopentenyl pyrophosphate (IPP) for rubber biosynthesis in the laticifers [6, 7]. We therefore characterized the key enzymes of the MVA pathway and upstream reactions in *T. brevicorniculatum* latex [see Fig. 1].

In several previous studies, 3-hydroxy-methyl-glutaryl-CoA reductase (HMGR) has been identified as the rate-limiting enzyme in the MVA pathway [8–12]. Plant HMGRs are characterized by four domains: a variable cytosolic N-terminus, two endoplasmic reticulum transmembrane domains, a variable region between the transmembrane domains, and a cytosolic C-terminus containing the highly-conserved catalytic domain [13, 14]. Plant HMGRs are regulated at the post-transcriptional and post-translational levels, e.g. by phosphorylation and inhibition by their own N-terminus [15–18]. *T. brevicorniculatum* produces three HMGRs encoded by the genes *TbHMGR1*, *TbHMGR2* and *TbHMGR3*, with *TbHMGR1* predominantly expressed in the latex [19]. MVA pathway engineering in plants is challenging [12, 20]. The overexpression of endogenous key enzymes often fails to increase the yield of end products due to negative feedback or rate-limiting downstream steps, and the regulatory mechanisms therefore need to be characterized in more detail [12, 21].

Enzymes acting upstream of HMGR in the latex might act as additional rate-limiting steps for isoprenoid biosynthesis, including ATP citrate lyase (ACL) and acetoacetyl-CoA thiolase (AACT) [see Fig. 1]. Acetyl-CoA is required in many anabolic and catabolic pathways in plants and must be synthesized in every cell compartment [22]. ACL cleaves citrate into oxaloacetate and acetyl-CoA in the cytosol. It belongs to the thiokinase superfamily, which also includes succinyl-CoA synthetase (SCS) and citrate synthase (CS) among others [23]. SCS is localized in mitochondria and catalyzes the reversible conversion of succinyl-CoA to succinate and CoA. Therefore, ATP-binding and CoA-binding domains are conserved between SCS and ACL. *Arabidopsis thaliana* ACL is a 500 kDa heteromeric protein comprised of ACLA and ACLB subunits. AtACLA is very similar to the β-subunit of AtSCS, whereas AtACLB is more closely related to the α-subunit of AtSCS and to AtCS [24]. Transgenic *A. thaliana* plants expressing *AtACLA-1* antisense RNA have a severe dwarf phenotype which underlines the importance of this enzyme for anabolic processes [25]. Furthermore, the overexpression of an *A. thaliana* AtACL(AB) fusion protein in dandelion latex increased ACL activity and led to the accumulation of various isoprenoid end products, highlighting its ability to increase flux through the MVA pathway [26]. The effective shuttling of acetyl-CoA into the MVA pathway requires a Claisen-type condensation reaction between two acetyl-CoA molecules to form acetoacetyl-CoA, which is catalyzed by AACT. In plants, catabolic 3-ketoacyl-CoA thiolases (type I thiolases; EC 2.3.1.16) and anabolic acetoacetyl-CoA thiolases (type II thiolases; EC 2.3.1.9) belong to the thiolase superfamily [27]. The MVA pathway requires type II thiolases, and the corresponding enzymes have been characterized in *A. thaliana*, *H. brasiliensis* and *Medicago sativa*. In *A. thaliana*, the *AtAACT1* and *AtAACT2* genes have different spatiotemporal expression profiles and knockout phenotypes: *Ataact1* T-DNA knockout lines show normal growth and development whereas *Ataact2* T-DNA knockout lines are embryonic lethal [28]. Furthermore, *AtAACT2* RNAi lines are sterile and are characterized by a reduced cell size and cell number, indicating that *AtAACT2* is essential among others for sterol biosynthesis via the MVA pathway, which is pivotal for membrane biosynthesis. In *H. brasiliensis*, *HbAACT1* is predominantly expressed in the latex whereas *HbAACT2* and *HbAACT3* are mainly expressed in mature leaves [7]. In *M. sativa*, the key role of MsAACT1 in isoprenoid biosynthesis was shown by overexpression experiments, which resulted in higher thiolase activity and the accumulation of squalene [29].

The dandelion *ACL* and *AACT* genes have not been characterized in detail but may play a key role in isoprenoid biosynthesis as in other plants. We therefore identified *ACL* and *AACT* genes predominantly expressed in *T. brevicorniculatum* latex and characterized their spatiotemporal expression profiles. We also overexpressed the well-characterized *A. thaliana HMGR*, *ACL* and *AACT* genes in *T. brevicorniculatum* to increase flux through the MVA pathway predominantly in the latex. This allowed us to determine the impact of the three enzymes on the accumulation of isoprenoid end products such as triterpenes and polyisoprenes.

## Methods

### Plant material and cultivation conditions

*T. brevicorniculatum* seeds were obtained from the Botanical Garden Marburg (Germany) and were cultivated at 18 °C and 20 klux with a 16-h photoperiod in controlled growth chambers or in the greenhouse. Plants were cultivated in a pre-fertilized 1:1 mixture of standard soil (ED73 Einheitserde, Fröndenberg, Germany) and garden mold (Botanical Garden Münster, Germany). They were fed every 4 weeks with a commercial fertilizer according to the manufacturer’s recommendations (Hakaphos Plus, Compo GmbH, Münster, Germany). *Arabidopsis thaliana* ecotype Columbia (Col-0) seeds were acquired from the Nottingham Arabidopsis Stock Centre (University of Nottingham, Loughborough, United Kingdom, http://arabidopsis.info) and cultivated as stated above. Seeds of *Nicotiana benthamiana* laboratory isolate (LAB) [30] were obtained from the Sainsbury Laboratory (John Innes Centre, Norwich, United Kingdom) and cultivated as stated above.

### RNA extraction and cDNA synthesis

Total RNA was extracted from *T. brevicorniculatum* root latex using the RNeasy Lipid Tissue Kit (Qiagen, Hilden, Germany), and from *T. brevicorniculatum* root, peduncle, leaf and flower tissues using the innuPREP RNA Mini Kit (Analytik Jena, Jena, Germany), each according to the manufacturer’s instructions. Full-length cDNA was synthesized from 500 ng total RNA using PrimeScript RT Master Mix (TaKaRa, Clontech, Saint-Germain-en-Laye, France) according to the manufacturer’s instructions. Full-length cDNA sequences were obtained by standard RACE procedures. Oligonucleotide sequences are shown in Additional file 1 [see Additional file 1].

### Cloning and transformation procedures

Expression vector pLab12.10-pREF-AtACL(AB) which contained the promoter of the rubber elongation factor (REF) was prepared as previously described [26]. The AtHMGR1(S408A) catalytic domain with a mutated phosphorylation site at position 408 was amplified from *A. thaliana* leaf cDNA using primers Athmgrc1ATG-PciI-fwd and Athmgrc1(S408A)-XbaI-rev, digested with PciI/XbaI and inserted at the NcoI/XbaI sites of pLab12.1pREF [5] resulting in final vector pLab12.1pREF-Athmgrc1(S408A). The same fragment was inserted into the pCambia1305.1 vector as previously described [19]. Expression of heterologous AtHMGR variants pCambia1305.1-Athmgrc1 and pCambia1305.1-Athmgrc1(S408A) in *Nicotiana benthamiana* was carried out according to van Deenen et al. [19]. The *Agrobacterium tumefaciens* strain GV3101pMP90RK used for infiltration was obtained from DNA Cloning Service e.K. (Hamburg, Germany). The AtAACT2 sequence was amplified from the same source using primers AtAACT2-SalI-fwd and AtAACT2-XbaI-rev, digested with SalI/XbaI and inserted at the XhoI/XbaI sites of the same plasmid resulting in the final vector pLab12.1pREF-AtAACT2. An expression vector containing both sequences was prepared by removing the pREF-Athmgrc1(S408A) cassette from pLab12.1pREF-Athmgrc1(S408A) using HindIII/BamHI and transferring it to the PmeI site in pLab12.1pREF-AtAACT2 to yield the final vector pLab12.1-pREF-AtAACT2-pREF-Athmgrc1(S408A). The transformation of *T. brevicorniculatum* by *Agrobacterium tumefaciens* strain EHA105 provided by Beth Hood, Prodigene Inc., College Station, TX, USA [31] was carried out as previously described [32]. The constructs pLab12.1pREF-Athmgrc1(S408A) and pLab12.1pREF-AtAACT2 were introduced into wild-type *T. brevicorniculatum* plants, whereas pLab12.1pREF-AtAACT2-pREF-Athmgrc1(S408A) was introduced into the transgenic *T. brevicorniculatum* line TbAB1 expressing the pREF-AtACL(AB) construct [26]. Oligonucleotide sequences are shown in Additional file 1.

### Quantitative RT-PCR (qRT-PCR)

Quantitative RT-PCR analysis was carried out as previously described [26] with slight modifications. Latex was harvested from 8-week-old roots of wild-type and transgenic *T. brevicorniculatum* plants for RNA isolation, cDNA synthesis and qRT-PCR using the KAPA SYBR® FAST qPCR Master Mix (Peqlab, Erlangen, Germany) in a Bio-Rad iCycler real-time PCR system. Three different *T. brevicorniculatum* reference genes were included in the qRT-PCR analysis: The glyceraldehyde-3-phosphate dehydrogenase (*TbGAPDH*) and the elongation factor-1 alpha (*TbEF1α*) as already described [26, 33], as well as the ribosomal protein L27 (*TbRP*) based on an expressed sequence tag (EST) (GenBank GO664824) of the *T. koksaghyz* root cDNA library. The relative expression levels of each target gene were calculated using Bio-Rad CFX Manager 3.1 software (Bio-Rad Laboratories Inc., Hercules, USA). All oligonucleotide sequences for the reference, endogenous and transgene expression analysis are shown in Additional file 1. Primer efficiencies, amplification factors and usage of formamide for enhancing primer specificity are shown in Additional file 2.

### SDS-PAGE and western blot analysis

*Taraxacum brevicorniculatum* protein was extracted and analyzed as previously described [26] with slight modifications. The rubber extraction buffer contained 100 mM Tris (pH 7.5), 350 mM sorbitol, 10 mM NaCl, 5 mM MgCl_2_, and 5 mM dithiothreitol (DTT). A rabbit antibody against *A. thaliana* ACLB was kindly provided by Yves Poirier (Department of Plant Molecular Biology, Université de Lausanne, Switzerland) and was diluted 1:2000 before use. A rabbit antibody against the *A. thaliana* HMGR1 catalytic domain was kindly provided by Hubert Schaller (Institut de Biologie Moléculaire des Plantes, Université de Strasbourg, France) and diluted 1:7500 before use. Both were detected with a secondary alkaline phosphatase (AP)-conjugated goat anti-rabbit IgG antibody diluted 1:10,000 before use. A mouse antibody against *A. thaliana* AACT2 was kindly provided by Basil Nikolau (Roy J. Carver Department of Biochemistry, Biophysics and Molecular Biology, Iowa State University, USA) and was diluted 1:1000 before use as previously described [28]. This was detected with a secondary AP-conjugated goat anti-mouse IgG antibody diluted 1:10,000 before use.

### Analysis of enzyme activity

ACL activity in dandelion latex from 8-week-old roots of *T. brevicorniculatum* plants was determined as previously described [26]. AACT activity was determined by measuring the reduction of 5,5′-dithiobis-(2-nitrobenzoic acid) (DTNB) by free CoA as previously described [34] with slight modifications. We incubated 5 μg of crude protein extracted from the latex of 8-week-old roots of *T. brevicorniculatum* plants in 95 μl reaction buffer (50 mM Tris/HCl, 0.5 M EDTA, 10 mM acetyl-CoA, 10 mM DTNB, pH 8.0) and measured the reduction of DTNB at 412 nm with a Tecan-Infinite®200 Pro (Tecan Group Ltd., Männedorf, Switzerland). To measure HMGR activity, crude latex harvested from 8-week-old roots of *T. brevicorniculatum* plants was transferred to ice-cold rubber extraction buffer, and ~40 μg total protein was used in a [3-^14^C]HMG-CoA incorporation assay as previously described [32, 35].

### Staining root cross-sections with Nile red

*Taraxacum brevicorniculatum* roots were sliced with a razor blade and incubated in a Nile red staining solution (1 mg/ml Nile red in dimethylsulfoxide (DMSO) stock solution diluted 1:150 in double-distilled water) for 30 min. Stained root cross-sections were analyzed under a Leica MZ16 F fluorescence stereomicroscope (Leica Microsystems GmbH, Wetzlar, Germany) using the GFP Plants filter set (excitation filter 450–490 nm, barrier filter 500–550 nm) and visualized with Leica Application Suite X software.

### Chemical analysis

Triterpene and poly(*cis*-1,4-isoprene) levels from *T. brevicorniculatum* root material were quantified via gas chromatography/mass spectrometry (GC/MS) and ^1^H nuclear magnetic resonance spectroscopy (^1^H–NMR), respectively, as previously described [36]. Dolichol levels were determined as previously described [5] using 1-g-aliquots of freeze-dried and ground *T. brevicorniculatum* root material.

### Statistical analysis

Normal distributions of gene expression levels and enzymatic activities at *p* < 0.05 were assessed using the Kolmogorov-Smirnov test in OriginPro 2016 (OriginLab, Northhampton, USA). Normally distributed datasets were analyzed using ANOVA with the post-hoc Tukey’s honest significant difference test. Datasets which did not show a normal distribution and could not be transformed to be normally distributed were analyzed using the Mann-Whitney U test.

## Results and discussion

### Synthesis of the full-length *ACLA*, *ACLB* and *AACT* cDNA sequences from *T. brevicorniculatum* latex RNA

The *T. brevicorniculatum* genome has not yet been sequenced, so expressed sequence tag (EST) databases at GenBank prepared from either root material of its close relative *T. koksaghyz* or from *T. officinale* (tissue not specified at GenBank) were searched for *ACLA*, *ACLB* and *AACT* cDNA sequences. Two different *T. koksaghyz ACLA* ESTs (GenBank accession numbers DR401254 and DR402930) as well as one *T. officinale ACLA* EST (DY808625) were found which covered the 5′ end of *ACLA* open reading frames based on comparisons with the *A. thaliana ACLA* genes At1g10670, At1g60810 and At1g09430. One full-length cDNA sequence could be amplified by 3’RACE using *T. brevicorniculatum* root latex RNA as template. The 1272-bp *TbACLA1* cDNA (GenBank accession number KY765686) based on the overlapping ESTs DR401254 and DR402930 encoded a protein of 423 amino acids. Furthermore, two different *T. koksaghyz* ESTs (DR398498 and DR399293) were found with high similarity to the *A. thaliana ACLB* orthologs At3g06650 and At5g4946. By performing 5’RACE using the same template one full-length *TbACLB* cDNA (1827 bp, GenBank accession number KY765687) encoding a protein of 608 amino acids named TbACLB1 (based on EST DR398498) was produced. Finally, *T. koksaghyz* ESTs DR398619 and DR401218 showed high similarity to the *A. thaliana AACT* genes At5g47720 and At5g48230. One full-length cDNA was produced by 3’RACE again using *T. brevicorniculatum* root latex RNA as the template. The 1227-bp *TbAACT1* cDNA sequence (GenBank accession number KY765685) based on EST DR398619 encoded a protein of 408 amino acids.

The deduced polypeptides of full-length cDNAs were aligned in silico with known ACLs and AACTs from *A. thaliana*, *Oryza sativa* and *H. brasiliensis* using Clustal Omega (https://www.ebi.ac.uk/Tools/msa/clustalo/) [see Additional file 3]. The TbACLA1 amino acid sequence showed 79–85% identity to ACLAs from *A. thaliana* (AtACLA1–3; Q9SGY2.1, O22718.1 and O80526.1) and *O. sativa* (OsACLA1–3; Q53JY8.2, Q2QZ86.2 and Q2QNG7.1). As shown in Additional file 3 A, TbACLA1 contains the ACL-SCS family signature 3 motif (PROSITE accession number PS01217) at residues 270–294 as well as the residues Lys4, Lys58, Glu116 and Asp213, which are conserved in the ATP grasp domain [23, 24].

The TbACLB1 amino acid sequence showed 90–91% identity to AtACLB1 (Q9C522.1), AtACLB2 (Q9FGX1.1) and OsACLB1 (Q93VT8.1). The multiple sequence alignment suggested the presence of the above mentioned ACL-SCS family signature 3 motif at TbACLB1 residues 168–193, as well as the ACL-SCS family signature 1 motif (PROSITE accession number PS01216) at TbACLB1 residues 174–203 and the ACL-SCS family active site (PROSITE accession number PS00399) at TbACLB1 residues 259–275, including the His273 residue which is thought be phosphorylated by ATP [see Additional file 3 B]. This confirmed the homology between TbACLB1 and SCS α-subunits.

The TbAACT1 protein sequence showed 79% identity to HbAACT1 (BAF98276.1), 78% identity to AtAACT2 (NP_568694.2) and 75% identity to AtAACT1 (NP_199583.1). The multiple sequence alignment indicated the presence of thiolase signature 2 (PROSITE accession number PS00737) at TbAACT1 residues 354–370, and the thiolase active site (PROSITE accession number PS00099) at TbAACT1 residues 389–402, including residues involved in the thiolase reaction cycle [37] [see Additional file 3 C]. The greater sequence identity between TbAACT1 and AtAACT2 compared to the functionally redundant AtAACT1 [28] suggests that TbAACT1 is the dominant AACT isoform in *T. brevicorniculatum*.

### Transcriptional regulation of the *TbACLA*, *TbACLB* and *TbAACT* genes

**Fig. 2:**
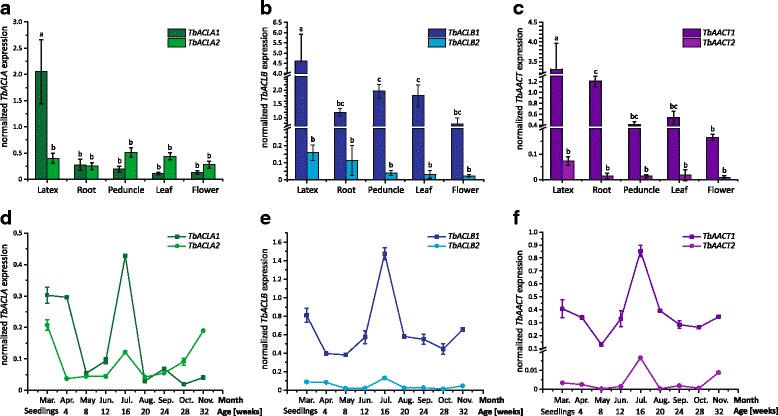
Spatial and temporal *ACLA/B* and *AACT* mRNA expression profile in wild-type *T. brevicorniculatum* plants determined by qRT-PCR. The corresponding mRNA levels were normalized against the constitutive genes elongation factor 1 α (*TbEF1α*), glyceraldehyde-3-phosphate dehydrogenase (*TbGAPDH*) and ribosomal protein L27 (*TbRP*) from *T. brevicorniculatum*. Bars represent standard errors of three independent wild-type plants per line. Normal distribution at *p* < 0.05 was assessed using the Kolmogorov-Smirnov test. Different letters indicate a significant difference (ANOVA with Tukey’s honest significant difference test (*p* < 0.01)). **a** *TbACLA1 and TbACLA2* **b** *TbACLB1* and *TbACLB2* and **c** *TbAACT1* and *TbAACT2* mRNA levels in latex, roots, peduncles, leaves and flowers of 10-week-old wild-type *T. brevicorniculatum* plants. **d** *TbACLA1* and *TbACLA2*, **e** *TbACLB1* and *TbACLB2* and **f** *TbAACT1* and *TbAACT2* mRNA levels in seedlings and root material of wild-type *T. brevicorniculatum* plants ranging in age from 4 to 32 weeks. Plants flowered in June

To determine whether the isolated *TbACLA1*, *TbACLB1* and *TbAACT1* genes encoded the dominant isoforms in the latex, qRT-PCR analyses were performed using RNA from the latex, root, peduncle, leaf and flower tissues of 10-week-old wild-type *T. brevicorniculatum* plants grown under greenhouse conditions. Furthermore, qRT-PCR primer pairs were included in the analysis that were based on the additional ESTs mentioned above which are partial cDNAs encoding a second isoform of ACLA, ACLB and AACT, named *TbACLA2* (based on DY808625), *TbACLB2* (DR399293) and *TbAACT2* (DR401218), respectively. For comparison, parallel qRT-PCR tests were carried out on the *TbHMGR1* and *TbHMGR2* genes, whose expression profiles have already been determined by RT-PCR [19]. *TbHMGR1* plays a pivotal role in the latex MVA pathway due to its expression predominantly in laticifers, whereas *TbHMGR2* expression is barely detected in latex but it is expressed more strongly than *TbHMGR1* in roots and leaves [19] [see Additional file 4]. Like *TbHMGR1*, we found that *TbACLA1*, *TbACLB1* and *TbAACT1* were predominantly expressed in the latex [see Fig. 2a–c]. The expression of these genes detected in all other tissues may reflect the presence of small amounts of latex in all parts of the dandelion plant, but might also reflect the necessity of a basal activity of theses enzymes to synthesize important MVA pathway-derived substances for regular plant development. Remarkably, an overall lower expression could be measured for *TbACLB2* and *TbAACT2* in all tissues analyzed compared to *TbACLB1* and *TbAACT1*, respectively, whereas *TbACLA2* showed a similar expression level in roots and even slightly increased expression levels in peduncle, leaf and flower tissues (3–4 fold) compared to *TbACLA1* indicating that TbACLA2 is an important isoform in these tissues. However, the tissue-specific qRT-PCR analyses clearly demonstrate that TbACLA1, TbACLB1 and TbAACT1 represent the dominant isoforms in latex and therefore appear to be highly relevant components of the *T. brevicorniculatum* latex MVA pathway and are likely to be required for the production of isoprenoid end products in this specialized tissue.

The potential co-regulated expression of *TbACLA*, *TbACLB* and *TbAACT* during dandelion development was investigated by cultivating *T. brevicorniculatum* wild-type plants outside the greenhouse from March to November. Total root material (containing the greatest amount of latex in relation to other tissues) was pooled from three individual plants per month and tested by qRT-PCR [see Fig. 2d–f]. In accordance to the tissue expression analysis, *TbACLB1* and *TbAACT1* comprised higher expression levels over the whole year in the dandelion root compared to *TbACLB2* and *TbAACT2*, respectively [see Fig. 2e and f], whereas in case of *TbACLA* the isoform *TbACLA2* seemed to play an important role at defined developmental stages such as in October and November [see Fig. 2d]. Overall, *TbACLA, TbACLB* and *TbAACT* displayed highly similar expression patterns with expression peaks at the seedling stage in March, and also in July and November indicative of tight co-regulation, as already reported for the *A. thaliana* ACLA and ACLB subunits [24]. Given that *A. thaliana ACLA* and *ACLB* mRNA accumulates in the epidermis and trichomes of expanding leaves as well as the epidermis of growing organs [24], *ACL* gene expression seems to be associated with (and upregulated during) plant growth, as supported by the developmental and growth defects observed in *A. thaliana ACL-*RNAi plants [25]. Therefore, ACL activity in the MVA pathway may be pivotal when large quantities of isoprenoids are required, e.g. for the synthesis of membrane sterols during development. The expression peaks in early development and in the summer were coordinated with the upregulation of *TbAACT* in March and July. This indicated that the *TbACL* and *TbAACT* genes are co-regulated to ensure the shuttling of precursors into the MVA pathway during these developmental stages which are characterized by rapid expansion of the total root volume and consequently also the differentiation of the specialized laticiferous cells. Interestingly, *TbACL* and *TbAACT* showed an additional expression peak in November, which is when dandelion plants accumulate high levels of polyisoprenes in the roots [36]. This might explain the highly active MVA pathway in the latex and therefore the additional upregulation of TbACL for providing precursors and TbAACT as the first enzymatic step in the cytosolic isoprenoid pathway.

Gene expression levels do not necessarily reflect the levels of AACT and HMGR enzyme activity due to downstream regulatory mechanisms such as phosphorylation, feedback inhibition and external stimuli [16, 17, 29, 38, 39]. Therefore, the posttranscriptional regulation of key MVA pathway enzymes will be an important additional aspect to address in future studies concerning the engineering of isoprenoid metabolism in dandelion latex.

### Overexpression of ACL, AACT and HMGR orthologs from *A. thaliana* in dandelion latex

Having identified and characterized the *T. brevicorniculatum* latex-specific isoforms of ACL and AACT, we investigated the effects of overexpressing these enzymes, using the better-characterized *A. thaliana* orthologs to reduce the likelihood of endogenous gene inhibition. We previously reported that overexpressing the *A. thaliana* ATP citrate lyase fusion construct *AtACL(AB)* enhanced ACL activity in *T. brevicorniculatum* [26]. Therefore, a transgenic *T. brevicorniculatum* line expressing *AtACL(AB)* from our previous study [26] was used here and served as a control line to complete the setup. The *A. thaliana* genome contains two paralogous AACT genes (*AtAACT1* and *AtAACT2*) although only *AtAACT2* is ubiquitously expressed [28]. The *AtAACT2* cDNA sequence was therefore cloned from *A. thaliana* leaf RNA and used for the stable transformation of wild-type *T. brevicorniculatum* plants.

**Fig. 3:**
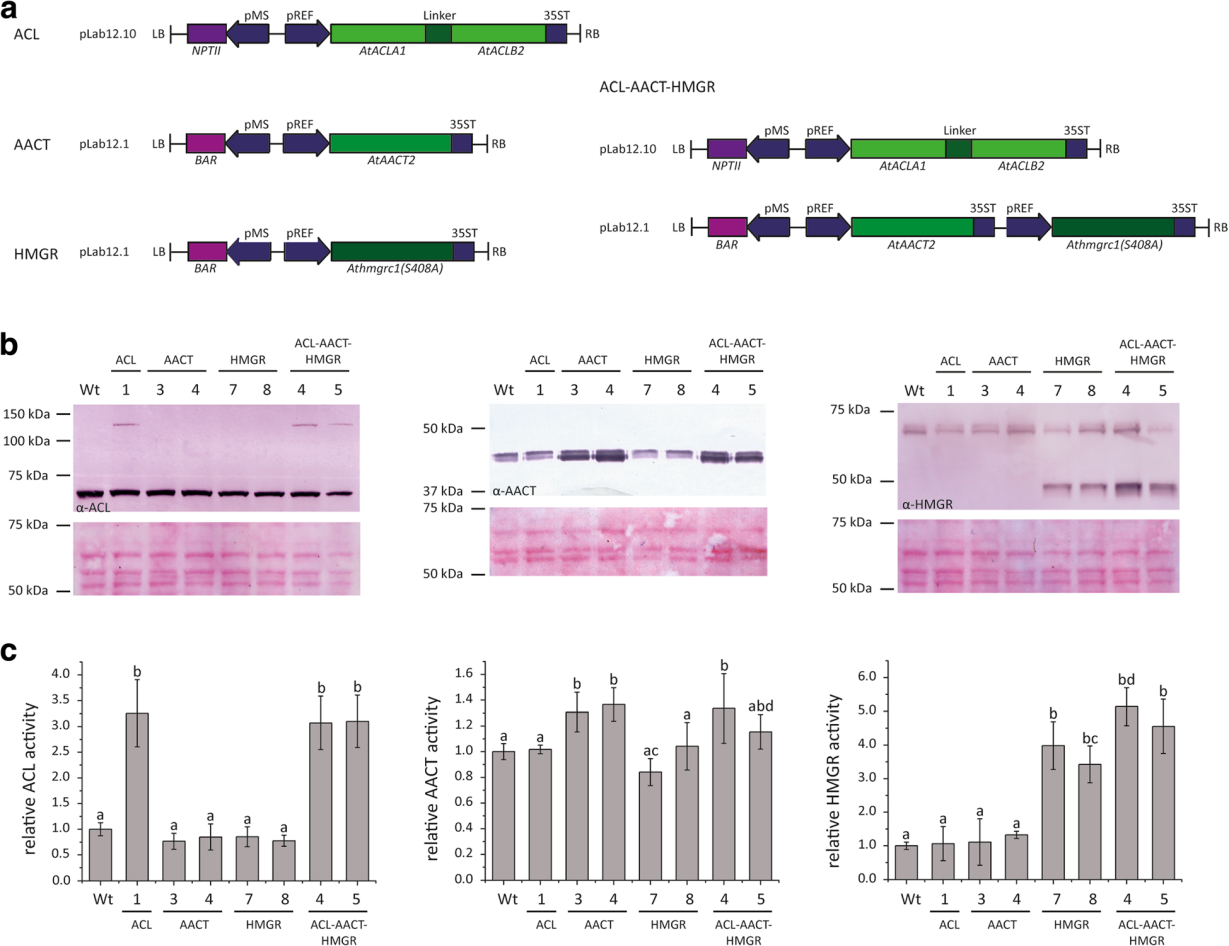
Expression of *A. thaliana* ACL, AACT and HMGR in transgenic *T. brevicorniculatum* plants. **a** T-DNA constructs for the overexpression of AtACL(AB), AtAACT2 and Athmgrc1(S408A) in *T. brevicorniculatum*. *NPTII*, kanamycin resistance gene; *BAR*, bialaphos resistance gene; pMS, mannopine synthase promoter; pREF, rubber elongation factor promoter; LB, left border; RB, right border. **b** Protein extracts from the latex of 8-week-old transgenic and wild-type (Wt) plants analyzed by SDS-PAGE and western blot (upper part) with antibodies against the AtACLB subunit (α-ACL), AtAACT2 (α-AACT) and HMGR (α-HMGR), or Ponceau S staining after protein transfer (lower part). Numbers on the left refer to molecular weight markers. **c** Relative ACL, AACT and HMGR enzymatic activity in the latex of 8-week-old transgenic and wild-type plants. The values of the wild-type plants were set to 1. Bars represent standard errors of three independent transgenic plants per line. Normal distribution at *p* < 0.05 was assessed using the Kolmogorov-Smirnov test. Different letters indicate a significant difference (ANOVA with Tukey’s honest significant difference test (*p* < 0.01))

HMGR catalyzes the rate-limiting step in the MVA pathway, and in *A. thaliana* the *AtHMGR1* gene is ubiquitously expressed [40]. The catalytic activity of plant HMGRs is inhibited by the N-terminal domain and by phosphorylation of the C-terminus, so we tested the activity of two different variants of AtHMGR1 in the heterologous *N. benthamiana* system. Athmgrc1(S408A) comprises the catalytic domain including the mutated phosphorylation site at position 408, and this was 2.5-fold more active than the wild-type catalytic domain Athmgrc1 [see Additional file 5]. *Athmgrc1(S408A)* was therefore used for the stable transformation of wild-type *T. brevicorniculatum* plants. The potential synergistic effect of *AtACL(AB)*, *AtAACT2* and *Athmgrc1(S408A)* overexpression was investigated by supertransforming transgenic *T. brevicorniculatum* plants expressing *AtACL(AB)* with constructs carrying *AtAACT2* and *Athmgrc1(S408A)*. All cDNAs were driven by the rubber elongation factor (REF) promoter to ensure preferential expression in the latex [41]. The constructs prepared for stable transformation are shown in Fig. 3a.

A set of 5–10 transgenic plant lines representing each transformation experiment was characterized by either RT-PCR or qRT-PCR in the T0 generation (data not shown). We then selected one line expressing the AtACL(AB) fusion construct (ACL-1), two lines overexpressing AtAACT2 (AACT-3 and AACT-4), two lines expressing Athmgrc1(S408A) (HMGR-7 and HMGR-8) and two lines expressing all three enzymes (ACL-AACT-HMGR-4 and ACL-AACT-HMGR-5) for detailed analysis in the T1 generation. Transgene expression in the latex harvested from roots of 8-week-old plants from all seven transgenic lines was confirmed by qRT-PCR [see Additional file 6 A]. The expression levels of the endogenous *TbACLA*, *TbAACT* and *TbHMGR1* genes were also compared to wild-type plants using the same technique [see Additional file 6 B]. No significant differences in endogenous gene expression levels were observed between wild-type plants and the transgenic lines, indicating that the overexpression of *A. thaliana* genes did not affect endogenous gene expression. We also measured the levels of the corresponding endogenous and heterologous proteins. Latex protein extracts obtained from roots were analyzed by western blot using antibodies specific for each enzyme [see Fig. 3b]. The anti-ACLB antibody revealed the presence of a 66 kDa band corresponding to the endogenous subunit in all lines and the presence of a 120 kDa band corresponding to the chimeric AtACL(AB) protein solely in the transgenic lines expressing the AtACL(AB) construct (ACL-1, ACL-AACT-HMGR-4 and ACL-AACT-HMGR-5). Likewise, the anti-AtAACT2 antibody revealed two protein bands of ~43 kDa and ~41 kDa, with the upper band corresponding to the endogenous TbAACT protein and the lower band corresponding to the AtAACT2 protein (the latter only present in transgenic lines overexpressing AACT: AACT-3, AACT-4, ACL-AACT-HMGR-4 and ACL-AACT-HMGR-5). Finally, the anti-HMGR antibody revealed one protein band of ~63 kDa representing the predominant endogenous latex isoform (TbHMGR1) and another of ~45 kDa representing the catalytic domain Athmgrc1(S408A), the latter present only in transgenic lines HMGR-7, HMGR-8, ACL-AACT-HMGR-4 and ACL-AACT-HMGR-5. These data confirmed that all transgenes were expressed in the latex as anticipated and were translated into the corresponding proteins without affecting endogenous protein levels.

Having confirmed the presence of the heterologous proteins and the absence of effects on endogenous proteins, we next measured the corresponding enzyme activities in latex crude protein extracts obtained from roots by determination of the conversion of [1,5-^14^C]citric acid to ^14^C–labeled acetyl-CoA (ACL), the reduction of DTNB by free CoA (AACT) and the conversion of [3-^14^C]HMG-CoA to [^14^C]mevalonolactone (HMGR) [see Fig. 3c]. ACL activity was significantly higher in the three transgenic lines expressing AtACL(AB) than the other lines (3.3-fold, 3.1-fold and 3.1-fold increases in lines ACL-1, ACL-AACT-HMGR-4 and ACL-AACT-HMGR-5, respectively). AACT activity was slightly higher in the four transgenic lines expressing the AtAACT2 construct (1.3-fold, 1.4-fold, 1.3-fold and 1.2-fold increases in lines AACT-3, AACT-4, ACL-AACT-HMGR4 and ACL-AACT-HMGR-5, respectively). Finally, all four transgenic lines expressing Athmgrc1(S408A) showed significantly higher levels of HMGR activity than the other lines (4.0-fold, 3.4-fold, 5.1-fold and 4.6-fold increases in lines HMGR-7, HMGR-8, ACL-AACT-HMGR-4 and ACL-AACT-HMGR-5, respectively). To our knowledge, this is the first report of the successful simultaneous overexpression of three functional transgenes in the latex of *T. brevicorniculatum*.

### Upregulation of the MVA pathway in *T. brevicorniculatum* latex enhances the accumulation of squalene, sterols, pentacyclic triterpenes and *cis*-1,4-isoprene

Whole roots from three 12-week-old plants per line were harvested and tested for their phenotypic and metabolic characteristics. The transgenic lines showed no overt phenotypic aberrations (representative images of one plant per line are compared to a wild-type plant in Additional file 7 A). Furthermore, there were no significant differences between the wild-type plants and transgenic lines in terms of root fresh weight or root dry weight [see Additional file 7 B and C]. The overexpression of heterologous AtACL(AB), AtAACT2 and Athmgrc1(S408A) in the latex therefore had no significant impact on the growth or development of *T. brevicorniculatum*. To ensure no major costs arise from this overexpression of transgenes, it would be necessary to measure the overall plant fitness by e.g. evaluating the seed production, quality and quantity.

**Fig. 4:**
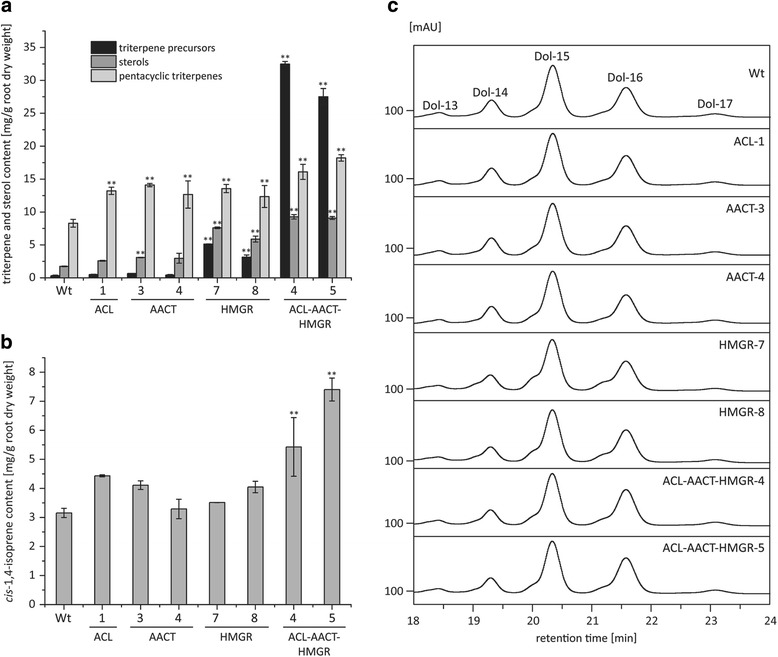
Influence of AtACL(AB), AtAACT2 and Athmgrc1(S408A) overexpression in dandelion latex on isoprenoid end products in 12-week-old *T. brevicorniculatum* roots. **a** Quantitative analysis of triterpene precursors (squalene and 2–3-oxidosqualene), sterols (campesterol, stigmasterol and sitosterol) and pentacyclic triterpenes (taraxasterol, α-amyrin, β-amyrin and lupeol) by GC/MS in freeze-dried roots of transgenic and wild-type (Wt) *T. brevicorniculatum* plants. Bars represent standard errors of three independent transgenic plants per line. Normal distribution at *p* < 0.05 was assessed using the Kolmogorov-Smirnov test. Asterisks represent significant differences relative to wild-type (ANOVA with Tukey’s honest significant difference test (*p* < 0.01)). **b** Quantitative analysis of *cis*-1,4-isoprene by ^1^H–NMR in freeze-dried roots of transgenic and wild-type *T. brevicorniculatum* plants. Bars represent standard errors of three independent transgenic plants per line. Normal distribution at *p* < 0.05 was assessed using the Kolmogorov-Smirnov test. Asterisks represent significant differences relative to wild-type (ANOVA with Tukey’s honest significant difference test (*p* < 0.01)). **c** Dolichol composition of freeze-dried root material determined by LC/MS. A mixture of dolichols (13–21 isoprene units) was used as a standard. One representative result for each transgenic line and a wild-type control is shown

Freeze-dried roots were used for triterpene extraction and GC/MS analysis because the quantitative analysis of triterpenes in latex tissue as described in Huber et al. [42] was not feasible for comparative analysis of wild-type and transgenic plant lines. The quantity of triterpene precursors was considerably higher in transgenic lines HMGR-7, HMGR-8, ACL-AACT-HMGR-4 and ACL-AACT-HMGR-5 than the other lines [see Fig. 4a]. Although the levels of the precursor 2,3-oxidosqualene were only slightly (1.7–1.9-fold) higher in the transgenic lines compared to wild-type plants, large amounts of squalene accumulated in the roots of the triple-transgenic lines (up to 32 mg/g root dry weight). Squalene accumulation was therefore investigated morphologically in cross-sections of wild-type and transgenic roots [see Additional file 8]. Nile red, which stains lipophilic molecules, revealed the presence of globules resembling lipid droplets in the root sections of all four transgenic lines. These globules were much more abundant in lines ACL-AACT-HMGR-4 and ACL-AACT-HMGR-5 than lines HMGR-7 and HMGR-8, and differed from laticifers in terms of size and position. These data suggest that excess squalene produced in these four transgenic lines is stored in lipid droplets outside the laticifers. These findings are supported by the observation of similar lipid droplets in tobacco (*Nicotiana tabacum*) leaves expressing full-length or truncated *HbHMGR1* [10, 15].

The average quantity of squalene accumulating in the roots of the triple-transgenic lines was 31.8 mg/g dry root weight for line ACL-AACT-HMGR-4 and 26.95 mg/g dry root weight for line ACL-AACT-HMGR-5. With the exception of a genetically engineered strain of *Pseudozyma* sp. (70 mg/g dry cell weight (DCW)) [43], this exceeds the highest squalene yields thus far achieved in microbes such as *Saccharomyces cerevisiae* (18.5 mg/g DCW), *Rhodopseudomonas palustris* (15.8 mg/g DCW) and *Schizochytrium mangrovei* (1.17 mg/g DCW) [44–46], but is lower than in plant tissues such as *Amaranthus* spp. seed oil (7% *w*/w) and olive oil (30% *w*/w) [47, 48]. However, the genetically engineered squalene content in transgenic *T. brevicorniculatum* roots by simultaneous overexpression of ACL, AACT and HMGR is within an industrially relevant range. Interestingly, the large amount of squalene present in the lipid droplets in the *T. brevicorniculatum* root does not appear to be toxic, which is consistent with experiments in *S. cerevisiae* showing that squalene accumulation is toxic only when lipid droplet formation is impaired [49]. The similar levels of 2,3-oxidosqualene in wild-type and transgenic plants despite the substantial differences in squalene levels indicate that the formation of 2,3-oxidosqualene catalyzed by squalene epoxidase might act as the main bottleneck between the MVA pathway and downstream isoprenoid biosynthesis.

We also analyzed the levels of other sterols, such as campesterol, stigmasterol and sitosterol. Overall, the quantity of sterols was higher in the roots of the transgenic lines than wild-type plants but the extent of sterol accumulation was dependent on the complement of transgenes. In the lines expressing ACL or AACT, the sterol levels were less than 2-fold higher than wild-type levels. In contrast, the sterol levels were 4.2-fold, 3.3-fold, 5.2-fold and 5.1-fold higher than wild-type in transgenic lines HMGR-7, HMGR-8, ACL-AACT-HMGR-4 and ACL-AACT-HMGR-5, respectively. This shows that the overexpression of ACL, AACT and HMGR increased flux through the MVA pathway in terms of IPP units directed towards sterol biosynthesis. Transgenic *T. brevicorniculatum* lines expressing HMGR produced the highest quantity of sterols, confirming that HMGR is the pivotal rate-limiting enzyme in the MVA pathway. Similarly, the overexpression of *HbHMGR1* in tobacco achieved a 6-fold increase in sterol levels [10] and the seed-specific expression of a truncated HbHMGR1 led to a 3.2-fold increase in seed sterol accumulation [15]. Our novel approach involved the simultaneous expression of three enzymes involved in the MVA pathway, achieving even higher sterol levels than the expression of HMGR alone.

Pentacyclic triterpenes such as taraxasterol, α-amyrin, β-amyrin and lupeol are also important triterpene components of *T. brevicorniculatum* latex because of their potential medical applications. For example, α-amyrin from *Bombax malabaricum* flowers is active against a range of bacteria and fungi [50] whereas α-amyrin, β-amyrin and lupeol display anti-inflammatory activities in mice and mammalian cell lines [51–53]. Taraxasterol has anti-allergic, anti-inflammatory and anti-carcinogenic effects [54–57]. These compounds are therefore valuable products in the biotechnology industry and efforts have already been undertaken to increase their yields, e.g. the addition of abiotic elicitors to root callus suspension cultures of *T. officinale*, a close relative of *T. brevicorniculatum*, to increase the production of taraxasterol [58]. In our transgenic lines overexpressing a single enzyme, the pentacyclic triterpene content increased by 1.5–1.7-fold compared to wild-type plants. In the triple-transgenic lines ACL-AACT-HMGR-4 and ACL-AACT-HMGR-5, the corresponding increase was 1.9–2.2-fold, suggesting that the overexpression of three MVA pathway enzymes has a synergistic effect. Such effects could therefore enhance the industrial production of pentacyclic triterpenes, especially in combination with the metabolic engineering of downstream pathways.

Next, we set out to determine the total *cis*-1,4-isoprene content in *T. brevicorniculatum* roots by ^1^H–NMR spectroscopy, which includes the analysis of polyisoprenes such as dolichols as well as high-molecular-weight long-chain poly(*cis*-1,4-isoprenes) that form the main constituent of natural rubber [see Fig. 4b]. All transgenic lines accumulated similar or slightly higher levels of *cis*-1,4-isoprene compared to wild-type plants, with lines ACL-1, ACL-AACT-HMGR-4 and ACL-AACT-HMGR-5 producing the highest levels (1.4-fold, 1.7-fold and 2.3-fold higher than wild-type, respectively). In these transgenic lines, IPP generated by the increase in flux through the MVA pathway was at least in part incorporated into *cis*-1,4-isoprene end products. We also investigated whether the greater abundance of precursors affected the chain length of known dolichols by liquid chromatography/mass spectrometry (LC/MS). However, no substantial differences in dolichol composition were observed between the wild-type and transgenic lines [see Fig. 4c].

The synthesis of polyisoprenes in the transgenic lines was not substantially affected by the overexpression of three key MVA pathway enzymes, which may reflect the bottleneck at the squalene epoxidase step. This step is therefore ideal for more detailed characterization in future experiments. Furthermore, the production of natural rubber in laticifers might not be dependent solely on the availability of IPP units, but also on the presence and activity of the enzymes that are directly linked to the rubber particles and thus build the rubber transferase complex [5]. These specialized end-product enzymes also represent useful targets for future genetic engineering approaches to increase the production of isoprenoid end products.

## Conclusions

We isolated full-length *ACL* and *AACT* cDNAs that are involved in production of isoprenoids in *T. brevicorniculatum* latex, namely *TbACLA*, *TbACLB* and *TbAACT*. The expression of the corresponding *A. thaliana* genes in *T. brevicorniculatum* latex, in addition to a derepressed version of HMGR (the rate-limiting enzyme in the MVA pathway) increased the activity of all three enzymes and affected the accumulation of isoprenoid end products. The heterologous overexpression of all three enzymes resulted in the accumulation of large amounts of squalene as well as higher levels of sterols, pentacyclic triterpenes and *cis*-1,4-isoprene in the roots of the transgenic lines. The synergistic effect of the three key enzymes could facilitate the industrial production of squalene or triterpenes. The yield could be improved substantially, especially in combination with the metabolic engineering of downstream enzymes.

## Additional files

Additional file 1: Sequences of oligonucleotides used for cloning and qRT-PCR. (PDF 81 kb)

Additional file 2: Primer efficiency, amplification factors and formamide usage for cDNA obtained from *T. brevicorniculatum* mRNA. The values were calculated using the Bio-Rad CFX Manager 3.1 software (Bio-Rad Laboratories Inc., Hercules, USA) and the qPCR primer efficiency calculator provided by Thermo Fisher Scientific (http://www.thermoscientificbio.com/webtools/qpcrefficiency/). (PDF 60 kb)

Additional file 3: In silico analysis of TbACLA1, TbACLB1 and TbAACT1 from *T. brevicorniculatum*. Alignments were created with Clustal Omega (https://www.ebi.ac.uk/Tools/msa/clustalo/). A: ACLA amino acid alignment with protein sequences from *A. thaliana* (At), *O. sativa* (Os) and *T. brevicorniculatum* (Tb). Sequences for AtACLA1 (Q9SGY2.1), AtACLA2 (O22718.1), AtACLA3 (O80526.1), OsACLA1 (Q53JY8.2), OsACLA2 (Q2QZ86.2) and OsACLA3 (Q2QNG7.1) were obtained from GenBank (https://www.ncbi.nlm.nih.gov/genbank/). Conserved residues representing the ATP grasp domain (Lys4, Lys48, Glu116 and Asp213) are shaded in light blue, and the ACL-SCS family signature 3 motif (PROSITE accession number PS01217) is shown in the yellow box. B: ACLB amino acid alignment with protein sequences from *A. thaliana* (At), *O. sativa* (Os) and *T. brevicorniculatum* (Tb). Sequences for AtACLB1 (Q9C522.1), AtACLB2 (Q9FGX1.1) and OsACLB1 (Q93VT8.1) were obtained from GenBank (https://www.ncbi.nlm.nih.gov/genbank/). The ACL-SCS family signature 3 motif (PROSITE accession number PS01217) and ACL-SCS signature 1 motif (PROSITE accession number PS01216) are shown in the green box, whereas the ACL-SCS family active site (PROSITE accession number PS00399) is shown in the pink box with the conserved (phosphorylated) residue His273 highlighted in light blue. C: AACT amino acid alignment with protein sequences from *A. thaliana* (At), *H. brasiliensis* (Hb) and *T. brevicorniculatum* (Tb). Sequences for AtAACT1 (NP_199583.1), AtAACT2 (NP_568694.2) and HbAACT1 (BAF98276.1) were obtained from GenBank (https://www.ncbi.nlm.nih.gov/genbank/). Conserved sites included thiolase signature 2 (PROSITE accession number PS00737, yellow box) and thiolase active site (PROSITE accession number PS00099, pink box). Residues involved in the thiolase reaction cycle are indicated in green [37]. (PDF 61 kb)

Additional file 4: Spatial *HMGR* expression profile in 10-week-old wild-type *T. brevicorniculatum* plants. Normalized *HMGR1* and *HMGR2* mRNA levels in latex, roots, peduncles, leaves and flowers were determined by qRT-PCR. The corresponding mRNA levels were normalized against the constitutive genes elongation factor 1 α (*TbEF1α*), glyceraldehyde-3-phosphate dehydrogenase (*TbGAPDH*) and ribosomal protein L27 (*TbRP*) from *T. brevicorniculatum*. Bars represent standard errors (*n* = three biological replicates). Normal distribution at *p* < 0.05 was assessed using the Kolmogorov-Smirnov test. Different letters indicate a significant difference (ANOVA with Tukey’s honest significant difference test (*p* < 0.01)). (TIFF 187 kb)

Additional file 5: Relative HMGR activity of two different Athmgrc1 variants in the heterologous *N. benthamiana* system. A: HMGR activity measured in *N. benthamiana* leaf extracts following the transient expression of Athmgrc1 constructs for 1 week. Athmgrc1, catalytic domain of AtHMGR1; Athmgrc1(S408A), catalytic domain of AtHMGR1 with a serine to alanine substitution at position 408. B: Protein extracts from *N. benthamiana* leaves transiently expressing Athmgrc1 variants analyzed by SDS-PAGE and western blot (upper part) using an antibody against HMGR. Ponceau S staining after protein transfer is shown below. Numbers on the left refer to molecular weight markers. (TIFF 116 kb)

Additional file 6: Normalized *ACL*, *AACT* and *HMGR* mRNA levels in transgenic and wild-type *T. brevicorniculatum* plants quantified by qRT-PCR. The corresponding mRNA levels were normalized against the constitutive gene elongation factor 1 α (*TbEF1α*) from *T. brevicorniculatum*. Bars represent standard errors (*n* = three biological replicates). A: *AtACLA1*, *AtAACT2* and *AtHMGR1* transgene mRNA levels in transgenic lines. No significant differences at *p* < 0.05 were detected among the transgenic lines using the Mann-Whitney U test. B: Endogenous *TbACLA*, *TbAACT* and *TbHMGR1* mRNA levels in all transgenic lines and wild-type (Wt) plants. No significant differences at *p* < 0.05 were detected between the wild-type plants and transgenic lines using the Mann-Whitney U test. (TIFF 1510 kb)

Additional file 7: Root morphology and weight of 12-week-old wild-type and transgenic *T. brevicorniculatum* plants. A: Cleaned roots were harvested and one representative plant from each line was photographed. Scale bar: 10 cm. B: Mean root fresh weight and C: mean root dry weight of harvested roots. Bars represent standard errors (*n* = three plants; asterisks indicate *n* = two plants). No significant differences at *p* < 0.05 were detected between wild-type (Wt) plants and transgenic lines using the Mann-Whitney U test. (TIFF 3459 kb)

Additional file 8: Root cross-sections of 12-week-old wild-type and transgenic *T. brevicorniculatum* plants. Staining was carried out with Nile red and one representative cross-section is shown for each line. Wt, wild-type; La, laticifer; Ld, lipid droplet. Scale bar: 250 μm.. (TIFF 5280 kb)

## Abbreviations

^1^H–NMR: ^1^H nuclear magnetic resonance spectroscopy
AACT: Acetoacetyl-CoA thiolase
ACL: ATP citrate lyase
At: *Arabidopsis thaliana*
CS: Citrate synthase
DCW: Dry cell weight
DMSO: Dimethylsulfoxide
DTNB: 5,5′-dithiobis-(2-nitrobenzoic acid)
DTT: Dithiothreitol
EF1α: Elongation factor-1 alpha
EST: Expressed sequence tag
GAPDH: Glyceraldehyde-3-phosphate dehydrogenase
GC/MS: Gas chromatography/mass spectrometry
Hb: *Hevea brasiliensis*
HMGR: 3-hydroxy-methyl-glutaryl-CoA reductase
IPP: Isopentenyl pyrophosphate
LAB: *Nicotiana benthamiana* laboratory isolate
LC/MS: Liquid chromatography/mass spectrometry
Ms.: *Medicago sativa*
MVA: Mevalonate
Os: *Oryza sativa*
qRT-PCR: Quantitative RT-PCR
REF: Rubber elongation factor
RP: Ribosomal protein L27
SCS: Succinyl-CoA synthetase
Tb: *Taraxacum brevicorniculatum*
Wt: Wild-type

## References

[1] Hagel JM, Yeung EC, Facchini PJ (2008). Got milk? The secret life of laticifers. Trends Plant Sci.

[2] Schulze Gronover C, Wahler D, Prüfer D. Natural Rubber Biosynthesis and Physic-Chemical Studies on Plant Derived Latex. 2011. p. 75–88.

[3] Takahashi S, Lee HJ, Yamashita S, Koyama T (2012). Characterization of cis-prenyltransferases from the rubber producing plant *Hevea brasiliensis* heterologously expressed in yeast and plant cells. Plant Biotechnol.

[4] Qu Y, Chakrabarty R, Tran HT, Kwon EJG, Kwon M, Nguyen TD (2015). A lettuce (*Lactuca sativa*) homolog of human Nogo-B receptor interacts with cis-prenyltransferase and is necessary for natural rubber biosynthesis. J Biol Chem.

[5] Epping J, van Deenen N, Niephaus E, Stolze A, Fricke J, Huber C (2015). A rubber transferase activator is necessary for natural rubber biosynthesis in dandelion. Nat Plants.

[6] Hepper CM, Audley BG (1969). The biosynthesis of rubber from ß-Hydroxy-ß-methylglutaryl- coenzyme a in *Hevea brasiliensis* latex. Biochem J.

[7] Sando T, Takeno S, Watanabe N, Okumoto H, Kuzuyama T, Yamashita A (2008). Cloning and characterization of the 2-C-methyl-D-erythritol 4-phosphate (MEP) pathway genes of a natural-rubber producing plant, *Hevea brasiliensis*. Biosci Biotechnol Biochem.

[8] Enjuto M, Lumbreras V, Marín C, Boronat A (1995). Expression of the Arabidopsis HMG2 Gene, encoding 3-Hydroxy-3-Methylglutaryl coenzyme a Reductase, is restricted to Meristematic and floral tissues. Plant Cell.

[9] Lumbreras V, Campos N, Boronat A (1995). The use of an alternative promoter in the *Arabidopsis thaliana* HMG1 gene generates an mRNA that encodes a novel 3-hydroxy-3-methylglutaryl coenzyme a reductase isoform with an extended N-terminal region. Plant J.

[10] Schaller H, Crausem B, Benveniste P, Chye M, Tan C, Song Y (1995). Expression of the *Hevea brasiliensis* (H.B.K.) Müll. Arg. 3-Hydroxy-3-Methylglutaryl-coenzyme a Reductase 1 in tobacco results in sterol overproduction. Plant Physiol.

[11] Suzuki M, Kamide Y, Nagata N, Seki H, Ohyama K, Kato H. Loss of function of 3-hydroxy-3-methylglutaryl coenzyme A reductase 1 ( HMG1 ) in Arabidopsis leads to dwar ® ng, early senescence and male sterility, and reduced sterol levels. Plant J 2004;1:750–761. 10.1111/j.1365-313x.2004.02003.x 14871314

[12] Liao P, Hemmerlin A, Bach TJ, Chye M-L (2016). The potential of the mevalonate pathway for enhanced isoprenoid production. Biotechnol Adv.

[13] Campos N, Boronat A (1995). Targeting and topology in the membrane of plant 3-hydroxy-3-methylglutaryl coenzyme a reductase. Plant Cell.

[14] Leivar P, González VM, Castel S, Trelease RN, López-Iglesias C, Arró M (2005). Subcellular localization of Arabidopsis 3-Hydroxy-3-Methylgluraryl-coenzyme a Reductase. Plant Physiol.

[15] Harker M, Holmberg N, Clayton JC, Gibbard CL, Wallace AD, Rawlins S (2003). Enhancement of seed phytosterol levels by expression of an N-terminal truncated *Hevea brasiliensis* (rubber tree) 3-hydroxy-3-methylglutaryl-CoA reductase. Plant Biotechnol J.

[16] Dale S, Becerra B, Morrice NG, Boronat A, Hardie DG, Ferrer A (1995). Bacterial expression of the catalytic domain of 3-hydroxy-3-methylglutaryl-CoA reductase (isoform HMGR1) from *Arabidopsis thaliana*, and its inactivation by phosphorylation at Ser577 by *Brassica oleracea* 3-hydroxy-3-methylglutaryl-CoA reductase kinase. Eur J Biochem.

[17] Leivar P, Antolín-Llovera M, Ferrero S, Closa M, Arró M, Ferrer A (2011). Multilevel control of Arabidopsis 3-hydroxy-3-methylglutaryl coenzyme a reductase by protein phosphatase 2A. Plant Cell.

[18] Guo D, Yi H-Y, Li H-L, Liu C, Yang Z-P, Peng S-Q (2015). Molecular characterization of HbCZF1, a *Hevea brasiliensis* CCCH-type zinc finger protein that regulates hmg1. Plant Cell Rep.

[19] van Deenen N, Bachmann A-L, Schmidt T, Schaller H, Sand J, Prüfer D (2012). Molecular cloning of mevalonate pathway genes from Taraxacum brevicorniculatum and functional characterisation of the key enzyme 3-hydroxy-3-methylglutaryl-coenzyme a reductase. Mol Biol Rep.

[20] Rodríguez-Concepción M, Boronat A (2015). Breaking new ground in the regulation of the early steps of plant isoprenoid biosynthesis. Curr Opin Plant Biol.

[21] Lange I, Poirier BC, Herron BK, Lange BM (2015). Comprehensive assessment of transcriptional regulation facilitates metabolic engineering of Isoprenoid accumulation in Arabidopsis. Plant Physiol.

[22] Xing S, Poirier Y (2012). The protein acetylome and the regulation of metabolism. Trends Plant Sci.

[23] Sánchez LB, Galperin MY, Müller M (2000). Acetyl-CoA synthetase from the amitochondriate eukaryote *Giardia lamblia* belongs to the newly recognized superfamily of acyl-CoA synthetases (nucleoside diphosphate-forming). J Biol Chem.

[24] Fatland BL, Ke J, Anderson MD, Mentzen WI, Cui LW, Allred CC (2002). Molecular characterization of a heteromeric ATP-citrate lyase that generates cytosolic acetyl-coenzyme a in Arabidopsis. Plant Physiol.

[25] Fatland BL, Nikolau BJ, Wurtele ES (2005). Reverse genetic characterization of Cytosolic acetyl-CoA generation by ATP-citrate Lyase in Arabidopsis. Plant Cell.

[26] Xing S, van Deenen N, Magliano P, Frahm L, Forestier E, Nawrath C (2014). ATP citrate lyase activity is post-translationally regulated by sink strength and impacts the wax, cutin and rubber biosynthetic pathways. Plant J.

[27] Dyer JH, Maina A, Gomez ID, Cadet M, Oeljeklaus S, Schiedel AC (2009). Cloning, expression and purification of an acetoacetyl CoA thiolase from sunflower cotyledon. Int J Biol Sci.

[28] Jin H, Song Z, Nikolau BJ (2012). Reverse genetic characterization of two paralogous acetoacetyl CoA thiolase genes in Arabidopsis reveals their importance in plant growth and development. Plant J.

[29] Soto G, Stritzler M, Lisi C, Alleva K, Pagano ME, Ardila F (2011). Acetoacetyl-CoA thiolase regulates the mevalonate pathway during abiotic stress adaptation. J Exp Bot.

[30] Bally J, Nakasugi K, Jia F, Jung H, Ho S, Wong M (2015). The extremophile Nicotiana Benthamiana has traded viral defence for early vigour. Nat Plants.

[31] Hood E, Gelvin S, Melchers L, Hoekema A (1993). New Agrobacterium helper plasmids for gene transfer to plants. Transgenic Res.

[32] Post J, van Deenen N, Fricke J, Kowalski N, Wurbs D, Schaller H (2012). Laticifer-specific cis-prenyltransferase silencing affects the rubber, triterpene, and inulin content of Taraxacum brevicorniculatum. Plant Physiol.

[33] Fricke J, Hillebrand A, Twyman RM, Prufer D, Schulze GC (2013). Abscisic acid-dependent regulation of small rubber particle protein Gene expression in Taraxacum brevicorniculatum is mediated by TbbZIP1. Plant Cell Physiol.

[34] Muoio DM, Noland RC, Kovalik J-P, Seiler SE, Davies MN, DeBalsi KL (2012). Muscle-specific deletion of Carnitine Acetyltransferase compromises glucose tolerance and metabolic flexibility. Cell Metab.

[35] Bach TJ, Lichtenthaler HK (1983). Inhibition by mevinolen of plant growth, sterol formation and pigment accumulation. Physiol Plant.

[36] Stolze A, Wanke A, van Deenen N, Geyer R, Prüfer D, Schulze GC. Development of rubber-enriched dandelion varieties by metabolic engineering of the inulin pathway. Plant Biotechnol J. 2016; doi:10.1111/pbi.12672. 10.1111/pbi.12672 PMC5425391 27885764

[37] Haapalainen AM, Meriläinen G, Pirilä PL, Kondo N, Fukao T, Wierenga RK (2007). Crystallographic and kinetic studies of human mitochondrial Acetoacetyl-CoA Thiolase: the importance of potassium and chloride ions for its structure and function. Biochemistry.

[38] Ruderman S, Kongsawadworakul P, Viboonjun U, Mongkolporn O, Chrestin H (2012). Mitochondrial/Cytosolic acetyl CoA and rubber biosynthesis genes expression in *Hevea brasiliensis* latex and rubber yield. Kasetsart J (Nat Sci).

[39] Rodríguez-Concepción M, Forés O, Martínez-García JF, González V, Phillips MA, Ferrer A (2004). Distinct light-mediated pathways regulate the biosynthesis and exchange of Isoprenoid precursors during Arabidopsis seedling development. Plant Cell.

[40] Enjuto M, Balcells L, Campos N, Caelles C, Arró M, Boronat A (1994). *Arabidopsis thaliana* Contains two differentially expressed 3-hydroxy-3-methylglutaryl-CoA reductase genes, which encode microsomal forms of the enzyme. Proc Natl Acad Sci U S A.

[41] Laibach N, Hillebrand A, Twyman RM, Prüfer D, Schulze GC (2015). Identification of a Taraxacum brevicorniculatum rubber elongation factor protein that is localized on rubber particles and promotes rubber biosynthesis. Plant J.

[42] Huber M, Triebwasser-Freese D, Reichelt M, Heiling S, Paetz C, Chandran JN (2015). Identification, quantification, spatiotemporal distribution and genetic variation of major latex secondary metabolites in the common dandelion (*Taraxacum officinale* agg.). Phytochemistry.

[43] Chang M-H, Kim H-J, Jahng K-Y, Hong S-C (2008). The isolation and characterization of Pseudozyma sp. JCC 207, a novel producer of squalene. Appl. Microbiol. Biotechnol.

[44] Mantzouridou F, Tsimidou MZ (2010). Observations on squalene accumulation in *Saccharomyces cerevisiae* due to the manipulation of HMG2 and ERG6. FEMS Yeast Res.

[45] Xu W, Chai C, Shao L, Yao J, Wang Y (2016). Metabolic engineering of Rhodopseudomonas palustris for squalene production. J Ind Microbiol Biotechnol.

[46] Yue C-J, Jiang Y (2009). Impact of methyl jasmonate on squalene biosynthesis in microalga Schizochytrium mangrovei. Process Biochem.

[47] Bondioli P, Mariani C, Lanzani A, Fedeli E, Muller A (1993). Squalene recovery from olive oil deodorizer distillates. J Am Oil Chem Soc.

[48] He H-P, Corke H (2003). Oil and Squalene in Amaranthus grain and leaf. J Agric Food Chem.

[49] Valachovic M, Garaiova M, Holic R, Hapala I (2016). Squalene is lipotoxic to yeast cells defective in lipid droplet biogenesis. Biochem Biophys Res Commun.

[50] El-Hagrassi AM, Ali MM, Osman AF, Shaaban M (2011). Phytochemical investigation and biological studies of *Bombax malabaricum* flowers. Nat Prod Res.

[51] Wu C-R, Hseu Y-C, Lien J-C, Lin L-W, Lin Y-T, Ching H (2010). Triterpenoid contents and anti-inflammatory properties of the methanol extracts of Ligustrum species leaves. Molecules.

[52] Okoye NN, Ajaghaku DL, Okeke HN, Ilodigwe EE, Nworu CS, FBC O (2014). Beta-Amyrin and alpha-amyrin acetate isolated from the stem bark of Alstonia Boonei display profound anti-inflammatory activity. Pharm Biol.

[53] Romero-Estrada A, Maldonado-Magaña A, González-Christen J, Bahena SM, Garduño-Ramírez ML, Rodríguez-López V (2016). Anti-inflammatory and antioxidative effects of six pentacyclic triterpenes isolated from the Mexican copal resin of Bursera copallifera. BMC Complement Altern Med.

[54] Liu J, Xiong H, Cheng Y, Cui C, Zhang X, Xu L (2013). Effects of taraxasterol on ovalbumin-induced allergic asthma in mice. J Ethnopharmacol.

[55] Wang S, Wang Y, Liu X, Guan L, Yu L, Zhang X (2016). Anti-inflammatory and anti-arthritic effects of taraxasterol on adjuvant-induced arthritis in rats. J Ethnopharmacol.

[56] Zhang X, Xiong H, Liu L (2012). Effects of taraxasterol on inflammatory responses in lipopolysaccharide-induced RAW 264.7 macrophages. J Ethnopharmacol.

[57] Takasaki M, Konoshima T, Tokuda K, Masuda K, Arai Y, Shiojima K (1999). Anti-carcinogenic activity of Taraxacum plant. II. Biol Pharm Bull.

[58] Sharma K, Zafar R (2016). Optimization of methyl jasmonate and β-cyclodextrin for enhanced production of taraxerol and taraxasterol in (*Taraxacum officinale* Weber) cultures. Plant Physiol Biochem.

