# Supplementary material for: Isoprenoid biosynthesis in dandelion latex is enhanced by the overexpression of three key enzymes involved in the mevalonate pathway
Source: BMC Plant Biol. 2017 May 22;17:88. doi: 10.1186/s12870-017-1036-0 (PMC5441070; doi:10.1186/s12870-017-1036-0)
Supplement: Supplementary file 1 — Sequences of oligonucleotides used for cloning and qRT-PCR. (PDF 81 kb) [file 12870_2017_1036_MOESM1_ESM.pdf]

Oligo	Sequence (5'→3')
	**full-length cDNA isolation**
TbAACT-3'RACE	CATGAAAGACGGGTTGTGGGA
TbAACT-fwd	AAAATGGCTCCAGCTGCGGC
TbAACT-rev	AAATCAAACAAGCTCTACTAC
TbACLA-3’RACE	AGGAGAGCACTTGTAGTAGGA
TbACLA-for	ATGGCACGCAAGATCCGAGAG
TbACLA-rev	AAATGCTTAAGCAGAAACCGTGATGC
TbACLB-5’RACE	ACCAATAGTCAACAAACCGGAAACAAGGCT
TbACLB-for	ATGGCGACTGGACAAATCTTCTCC
TbACLB-rev	TCACTTGGTGTAAAGAACGTCTTC
	**cloning procedures**
AtAACT2-SalI-fwd	AAAGTCGACACCATGGCCCATACATCAGAATCT
AtAACT2-XbaI-rev	AAATCTAGATCAAAGGAGCTCAAGTAGAGC
Athmgrc1ATG-PciI-fwd	AAAACATGTCAGAGGAAGACGAGGAGATTGTGA
Athmgrc1(S408A)-XbaI-rev	AAATCTAGATCATGTTGTTGTTGTTGTCGTTGTCGTTGCTCCAGAGATGTC TCGGCTGGCTCT
	**qRT-PCR**
AtAACT2-fw-realtime	GTCTATAACGACTGTGGGATGG
AtAACT2-rv-realtime	GTGAAGGCGCCAGCTTCC
AtACLA1-fw-realtime	CTCTCGTCTGAGAAGCTGGTG
AtACLA1-rv-realtime	GTCCTTTGCATCCACTCATCTCT
AtHMGR1-fw-realtime	GAGGAACACAGCTTGCATCTC
AtHMGR1-rv-realtime	CTAAAACTGCTCCGGCTACGA
TbAACT1-fw-realtime	CATGAAAGACGGGTTGTGGGA
TbAACT1-rv-realtime	TCAAAGGCCCCACTATCTCTG
TbAACT2-fw-realtime	CCGATTCCAAAATGGGAACAT
TbAACT2-rv-realtime	GAAAAAGCGCCACTGTCTCTA
TbACLA1-fw-realtime	GGTTATCTTCATCCAAACTCGTCG
TbACLA1-rv-realtime	GTCCTTGACATCCTCCCATTAC
TbACLA2-fw-realtime	GGCTCTCATCATCCAAATTAGTC
TbACLA2-rv-realtime	GACCTTTGCATCCTCCCATTTC
TbACLB1-fw-realtime	GGCATTCGAGTACCCGGAATT
TbACLB1-rv-realtime	TGAAAGCGTGTAAGTCTCCACTT
TbACLB2-fw-realtime	TATCCGTGTGCCTGGAATTGGT
TbACLB2-rv-realtime	GAGAGAGTGTAGGTTTCTACTTC
TbEF1alpha-fw-realtime	CGAGAGATTCGAGAAGGAAGC
TbEF1alpha-rv-realtime	CTGTGCAGTAGTACTTGGTGG
TbGAPDH-fw-realtime	TTGGAATTGTCGAGGGTCTC

TbGAPDH-rv-realtime	TGCTGCTAGGGATGATGTTG
TbHMGR1-fw-realtime	CCGGCGAATTTCTCAATCAGCA
TbHMGR1-rv-realtime	TCTAATCTTCTCGCGCCAACGT
TbHMGR2-fw-realtime	CAGTACGATGATCAGATA
TbHMGR2-rv-realtime	GACCTGATCTTTTCACGC
TbRP-fw-realtime	CGTCGATCTCAAGGATGTTGTC
TbRP-rv-realtime	GGAGCTTTGAGAAGAACCAACG
