# Supplementary material for: Isoprenoid biosynthesis in dandelion latex is enhanced by the overexpression of three key enzymes involved in the mevalonate pathway
Source: BMC Plant Biol. 2017 May 22;17:88. doi: 10.1186/s12870-017-1036-0 (PMC5441070; doi:10.1186/s12870-017-1036-0)
Supplement: Supplementary file 2 — Primer efficiency, amplification factors and formamide usage for cDNA obtained from T. brevicorniculatum mRNA. The values were calculated using the Bio-Rad CFX Manager 3.1 software (Bio-Rad Laboratories Inc., Hercules, USA) and the qPCR primer efficiency calculator provided by Thermo Fisher Scientific (http://www.thermoscientificbio.com/webtools/qpcrefficiency/). (PDF 60 kb) [file 12870_2017_1036_MOESM2_ESM.pdf]

Oligo pair	Efficiency	Amplification factor (66°C)	Formamide (1:50 dilution)
AtAACT2-realtime	90.94%	1.91	no
AtACLA1-realtime	110.58%	2.11	no
AtHMGR1-realtime	109.47%	2.09	no
TbAACT1-realtime	108.24%	2.08	no
TbAACT2-realtime	123.75%	2.24	no
TbACLA1-realtime	110.07%	2.10	yes
TbACLA2-realtime	109.87%	2.10	yes
TbACLB1-realtime	100.33%	2.00	yes
TbACLB2-realtime	103.72	2.04	yes
TbEF1alpha-realtime	104.48%	2.04	no
TbGAPDH-realtime	107.14%	2.07	no
TbHMGR1-realtime	104.94%	2.05	no
TbHMGR2-realtime	105.91%	2.06	no
TbRP-realtime	105.44%	2.05	no
