# Supplementary material for: Isoprenoid biosynthesis in dandelion latex is enhanced by the overexpression of three key enzymes involved in the mevalonate pathway
Source: BMC Plant Biol. 2017 May 22;17:88. doi: 10.1186/s12870-017-1036-0 (PMC5441070; doi:10.1186/s12870-017-1036-0)
Supplement: Supplementary file 3 — In silico analysis of TbACLA1, TbACLB1 and TbAACT1 from T. brevicorniculatum. Alignments were created with Clustal Omega (https://www.ebi.ac.uk/Tools/msa/clustalo/). A: ACLA amino acid alignment with protein sequences from A. thaliana (At), O. sativa (Os) and T. brevicorniculatum (Tb). Sequences for AtACLA1 (Q9SGY2.1), AtACLA2 (O22718.1), AtACLA3 (O80526.1), OsACLA1 (Q53JY8.2), OsACLA2 (Q2QZ86.2) and OsACLA3 (Q2QNG7.1) were obtained from GenBank (https://www.ncbi.nlm.nih.gov/genbank/). Conserved residues representing the ATP grasp domain (Lys4, Lys48, Glu116 and Asp213) are shaded in light blue, and the ACL-SCS family signature 3 motif (PROSITE accession number PS01217) is shown in the yellow box. B: ACLB amino acid alignment with protein sequences from A. thaliana (At), O. sativa (Os) and T. brevicorniculatum (Tb). Sequences for AtACLB1 (Q9C522.1), AtACLB2 (Q9FGX1.1) and OsACLB1 (Q93VT8.1) were obtained from GenBank (https://www.ncbi.nlm.nih.gov/genbank/). The ACL-SCS family signature 3 motif (PROSITE accession number PS01217) and ACL-SCS signature 1 motif (PROSITE accession number PS01216) are shown in the green box, whereas the ACL-SCS family active site (PROSITE accession number PS00399) is shown in the pink box with the conserved (phosphorylated) residue His273 highlighted in light blue. C: AACT amino acid alignment with protein sequences from A. thaliana (At), H. brasiliensis (Hb) and T. brevicorniculatum (Tb). Sequences for AtAACT1 (NP_199583.1), AtAACT2 (NP_568694.2) and HbAACT1 (BAF98276.1) were obtained from GenBank (https://www.ncbi.nlm.nih.gov/genbank/). Conserved sites included thiolase signature 2 (PROSITE accession number PS00737, yellow box) and thiolase active site (PROSITE accession number PS00099, pink box). Residues involved in the thiolase reaction cycle are indicated in green [37]. (PDF 61 kb) [file 12870_2017_1036_MOESM3_ESM.pdf]

A

```
AtACLA3     MARKKIREYDSKRLLKEHLKRLANIDLQIRSAQVTESTDFTELTNQESWLSSTKLVVKPD     60
OsACLA3     MARKKIREYDSKRLLREHLKRLAAIDLHILSAQVTESTDFTELVNQEPWLSSMKLVVKPD     60
OsACLA2     MARKKIREYDSKRLLKEHLKRLAGIDLQILSAQVTQSTDFTELVNQQPWLSTMKLVVKPD     60
OsACLA1     MARKKIREYDSKRLLKEHLKRLAGIDLQILSAQVTQSTDFTELVNQQPWLSTMKLVVKPD     60
TbACLA1     MARKKIREYDSKRLVKEHYKRISGSELPIKSAQVTESTDFNELVQSEPWLSSSKLVVKPD     60
AtACLA1     MARKKIREYDSKRLVKEHFKRLSGKELPIRSVQINETTDLNELVEKEPWLSSEKLVVKPD     60
AtACLA2     MARKKIREYDSKRLVKEHFKRLSGQELPIRSVQINQETDLNELVEREPWLSSEKLVVKPD     60
            **************::** **::  :* * *.*:.: **:.**.: : ***: *******

AtACLA3     MLFGKRGKSGLVALKLDLAEVADFVKARLGTEVEMEGCKAPITTFIVEPFVPHDQEYYLS     120
OsACLA3     MLFGKRGKSGLVALNLDLAQVRQFVKERLGVEVEMGGCKAPITTFIVEPFVPHDQEYYLS     120
OsACLA2     MLFGKRGKSGLVALNLDIAQVKEFVKERLGVEVEMGGCKAPITTFIVEPFVPHDQEYYLS     120
OsACLA1     MLFGKRGKSGLVALNLDIAQVKEFVKERLGVEVEMGGCKAPITTFIVEPFVPHDQEYYLS     120
TbACLA1     MLFGKRGKSGLVALNLDLAQVAEFVKERLGKEVVMGGCQGPITTFIVEPFVPHNEEFYIN     120
AtACLA1     MLFGKRGKSGLVALKLDFADVATFVKERLGKEVEMSGCKGPITTFIVEPFVPHNEEYYLN     120
AtACLA2     MLFGKRGKSGLVALNLDFADVATFVKERLGKEVEMSGCKGPITTFIVEPFVPHNEEFYLN     120
            **************:**:*:*   *** *** ** * **:.*************::*:*:.

AtACLA3     IVSDRLGCTISFSECGGIEIEEENWDKVKTIFLPAEKSMTLEVCAPLIATLPLEVRAKIGN     180
OsACLA3     IVSERLGSTISFSECGGIEIEEENWDKVKTVFLPTEKAMTPDACAPLIATLPLEVRTKIGD     180
OsACLA2     IVSERLGSTISFSECGGIEIEEENWDKVKTIFLPTEKPMTPDACAPLIATLPLEARGKIGD     180
OsACLA1     IVSERLGSTISFSECGGIEIEEENWDKVKTIFLSTEKPMTPDACAPLIATLPLEARGKIGD     180
TbACLA1     IVSERLGCSISFSECGGIDIEEENWDKVKTIFLPTGVSLSQEICAPLVATLPLEFKSVIEQ     180
AtACLA1     VVSDRLGCSISFSECGGIEIEEENWDKVKTIFLPTGASLTPEICAPLVATLPLEIKAEIEE     180
AtACLA2     IVSDRLGCSISFSECGGIDIEEENWDKVKTITIPTGPSLTFEICAPLVATLPLEIKGELED     180
            :**:***.:*********:**********:  :  :   :: : ****:****** :  : :

AtACLA3     FIMGAFAVFQDLDFSFMEMNPFTLVDGEPFPLDMRGELDDTAAFKNF-NKWGDIEFPLPF     239
OsACLA3     FIRGVYSVFQDLDFSFLEMNPFTMVNGEPYPLDMRGELDDTAAFKNF-KKWGNIQFPLPF     239
OsACLA2     FIKGVFAVFQDLDFSFLEMNPFTIVNGEPYPLDMRGELDDTAAFKNF-KKWGNIEFPLPF     239
OsACLA1     FIKGVFAVFQDLDFSFLEMNPFTIVNGEPYPLDMRGELDDTAAFKTSRSKWGNIEFPLPF     240
TbACLA1     FITHIYSLFVDLDFTFLEMNPFTLVDGKPYPLDMRGELDDTAAFKNF-KKWGNIEFPMPF     239
AtACLA1     FIKVIFTLFQDLDFTFLEMNPFTLVDGSPYPLDMRGELDDTAAFKNF-KKWGDIEFPLPF     239
AtACLA2     FIQVIFTLFEDLDFTFLEMNPFTLVDGKPYPLDMRGELDDTAAFKNF-KKWGDIEFPMPF     239
            **   ::* ****:*:******:*:*.*:***************.  .***:*:**:**

AtACLA3     GRVLSSTENFIHGLDEKTSASLKFTVLNPKGRIWTMVAGGGASVIYADTVGDLGYASELG     299
OsACLA3     GRVLSPSESFIHELDEKTSSSLKFTVLNPKGRIWTMVAGGGASVIYADTVGDLGYASELG     299
OsACLA2     GRVLSSTEGFIHDLDEKTSASLKFTVLNPKGRIWTMVAGGGASVIYADTVGDLGYASELG     299
OsACLA1     GRVLSSTEGFIHDLDEKTSASLKFTVLNPKGRIWTMVAGG-----------------ELE     283
TbACLA1     GRVMSATESFIHGLDEKTSASLKFTVLNPKGRIWTMVAGGGASVIYADTVGDLGFANELG     299
AtACLA1     GRVMSPTESFIHGLDEKTSASLKFTVLNPKGRIWTMVAGGGASVIYADTVGDLGYASELG     299
AtACLA2     GRVMSSTESFIHGLDEKTSASLKFTVLNPKGRIWTMVAGGGASVIYADTVGDLGYASELG     299
            ***:*  :*.*** ******:*******************                 **

AtACLA3     NYAEYSGAPNEEEVLQYARVVIDCATTDPDGRKRALLIGGGIANFTDVAATFNGIIRALR     359
OsACLA3     NYAEYSGAPNEEEVLQYARVVLDCATADPDGRKRALLIGGGIANFTDVAATFSGIIRALR     359
OsACLA2     NYAEYSGAPNEEEVLQYARVVLDCATADPDGRKRALLIGGGIANFTDVGATFSGIIRALR     359
OsACLA1     NYAEYSGAPNEEEVLQYARVVLDCATADPDGRKRALLIGGGIANFTDVGATFSGIIRALR     343
TbACLA1     NYAEYSGAPNEEEVLQYARVVIDCATADPDGQRRALVVGGGIANFTDVAATFNGIIRAMK     359
AtACLA1     NYAEYSGAPKEDEVLQYARVVIDCATANPDGKSRALVIGGGIANFTDVAATFNGIIRALK     359
AtACLA2     NYAEYSGAPKEDEVLQYARVVIDCATANPDGKSRALVIGGGIANFTDVAATFNGIIRALK     359
            *********:*:*********:****::***: ***::**********.***.*****::

AtACLA3     EKETRLKASRMHIYVRRGGPNYQTGLARMRALGEELGVPLEVYGPEATMTGICKRAIDCI     419
OsACLA3     EKESKLKAARMNIYVRRGGPNYQTGLAKMRTLGAELGVPIEVYGPEATMTGICKQAIDCI     419
OsACLA2     EKESKLKAARMHIYVRRGGPNYQTGLAKMRKLGAELGVPIEVYGPEATMTGICKQAIECV     419
OsACLA1     EKESKLKAARMHIYVRRGGPNYQTGLAKMRKLGAELGVPIEVYGPEATMTGICKQAIECV     403
TbACLA1     EKVEKLKAASMHIYVRRGGPNYQRGLARMRALGAELGIPIEVYGPEATMTGICKQAIECI     419
AtACLA1     EKEAKLKAARMHIFVRRGGPNYQKGLAKMRALGDDIGVPIEVYGPEATMTGICKEAIQYI     419
AtACLA2     EKEAKLKAARMHIFVRRGGPNYQKGLAKMRSLGDEIGVPIEVYGPEATMTGICKEAIQYI     419
            **  :***: *:*:********* ***:** ** ::*:*:**************.**: :

AtACLA3     MLPDA        424
OsACLA3     MAEA-        423
OsACLA2     MAAA-        423
OsACLA1     MAAA-        407
TbACLA1     TVSA-        423
AtACLA1     TAAA-        423
AtACLA2     TAAA-        423
```

B

```
TbACLB1       MATGQIFSRTTQALFYNYKQLPIQRMLDFDFLCGRETPSVAGIINPGAEGFQKLFFGQEE     60
OsACLB1       MATGQIFSKTTQALFYNYKQLPIQRMLDFDFLCGRETPSVAGIINPGSDGFQKLFFGQEE     60
AtACLB2       MATGQLFSRTTQALFYNYKQLPVQRMLDFDFLCGRETPSVAGIINPGSEGFQKLFFGQEE     60
AtACLB1       MATGQLFSRNTQALFYNYKQLPIQRMLDFDFLCGRETPSVAGIINPGSEGFQKLFFGQEE     60
              *****:**:.************:************************::***********

TbACLB1       IAIPVHSTIEAACAAHPTADVFINFASFRSAAASSKLALKQPTIRVVAIIAEGVPESDTK     120
OsACLB1       IAIPVHPTIEAACNAHPTADVFINFASFRSAAASSMSALKQPTIRVVAIIAEGVPESDTK     120
AtACLB2       IAIPVHAAIEAACAAHPTADVFINFASFRSAAASSMAALKQPTIKVVAIIAEGVPESDTK     120
AtACLB1       IAIPVHAAIEAACAAHPTADVFINFASFRSAAASSMAALKQPTIKVVAIIAEGVPESDTK     120
              ****** :***** *********************  *******:***************

TbACLB1       ELISYARSNNKVVIGPATVGGIQAGAFKIGDTAGTIDNIIQCKLYRPGCVGFVSKSGGMS     180
OsACLB1       QLISYARANNKVIIGPATVGGIQAGAFKIGDTAGTIDNIIQCKLYRPGSVGFVSKSGGMS     180
AtACLB2       QLIAYARANNKVVIGPATVGGIQAGAFKIGDTAGTIDNIIQCKLYRPGSVGFVSKSGGMS     180
AtACLB1       QLIAYARANNKVIIGPATVGGVQAGAFKIGDTAGTIDNIIQCKLYRPGSVGFVSKSGGMS     180
              :**:***:****:********:**************************.***********

TbACLB1       NELYNTIARVTDGLYEGIAIGGDVFPGSTLSDHVLRFNNIPQIKMIVVLGELGGRDEYSL     240
OsACLB1       NEMYNTIARVTDGIYEGIAIGGDVFPGSTLSDHILRFNNIPQVKMMVVLGELGGKDEYSL     240
AtACLB2       NEMYNTVARVTDGIYEGIAIGGDVFPGSTLSDHILRFNNIPQIKMMVVLGELGGRDEYSL     240
AtACLB1       NEMYNTIARVTDGIYEGIAIGGDVFPGSTLSDHILRFNNIPQIKMVVVLGELGGRDEYSL     240
              **:***:******:*******************:********:**:********:*****

TbACLB1       VEALKSGKISKPVCAWVSGTCATLFKSEVQFGHAGAKSGGEMESAQAKNQALKDAGAVVP     300
OsACLB1       VEALKQGKVQKPVVAWVSGTCARLFKSEVQFGHAGAKSGGELESAQAKNQALKDAGAVVP     300
AtACLB2       VEALKEGKVNKPVVAWVSGTCARLFKSEVQFGHAGAKSGGEMESAQAKNQALIDAGAIVP     300
AtACLB1       VEAMKQGKVTKPVVAWVSGTCARLFKSEVQFGHAGAKSGGEMESAQAKNQALQDAGATVP     300
              ***:*.**: *** ******** ******************:********** **** **

TbACLB1       TSFESFETSIKETYEKLAEEGKIAPVKEITPPQIPEDLNSAIKSGKVRAPTHIISTISDD     360
OsACLB1       TSYEALETAIKETFEKLVEDGKISPVTEITPPPIPEDLKTAIKSGKVRAPTHIISTISDD     360
AtACLB2       TSFEALESAIKETFEKLVEEGKVSPIKEVIPPQIPEDLNSAIKSGKVRAPTHIISTISDD     360
AtACLB1       TSFEALEVAIKETFDKLVEEGKVSPIKEVTPPQIPEDLSSAIKSGKVRAPTHIISTISDD     360
              **:*::* :****::**.*:**::*:.*: ** *****.:********************

TbACLB1       RGEEPCYAGVPMSTIVEKGMGVGDVISLLWFKRSLPRYCTQFIEICIMLCADHGPCVSGA     420
OsACLB1       RGEEPCYAGVPMSTIIEQGYGVGDVISLLWFKRSLPRYCTQFIEMCIMLCADHGPCVSGA     420
AtACLB2       RGEEPCYAGVPMSSIIEQGYGVGDVISLLWFKRSLPRYCTKFIEICIMLCADHGPCVSGA     420
AtACLB1       RGEEPCYAGVPMSSIIEQGYGVGDVISLLWFKRSLPRYCTKFIEICIMLCADHGPCVSGA     420
              *************:*:*:* ********************:***:***************

TbACLB1       HNTIVTARAGKDLVSCLVSGLLTIGPRFGGAVDDAARYFKDAYDRNLTPYEFVESMKKKG     480
OsACLB1       HNSIVTARAGKDLVSSLVSGLLTIGPRFGGAIDDAARYFKDAYDRNLTPYEFVEGMKKKG     480
AtACLB2       HNTIVTARAGKDLVSSLVSGLLTIGPRFGGAIDDAARYFKDACDRNLTPYEFVEGMKKKG     480
AtACLB1       HNTIVTARAGKDLVSSLVSGLLTIGPRFGGAIDDAARYFKDACDRNLTPYEFVEGMKKKG     480
              **:************.***************:********** ***********.*****

TbACLB1       IRVPGIGHRIKRGDNRDKRVELLQLFARTNFPSTKYMEYAVEVETYTLSKSNNLVMNVDG     540
OsACLB1       IRVPGIGHRIKSRDNRDKRVQLLQKYAHTHFPSVKYMEYAVQVETYTLSKANNLVLNVDG     540
AtACLB2       IRVPGIGHRIKSRDNRDKRVELLQKFARSNFPSVKYMEYAVTVETYTLSKANNLVLNVDG     540
AtACLB1       IRVPGIGHRIKSRDNRDKRVELLQKFARSNFPAVKYMEYAVQVETYTLSKANNLVLNVDG     540
              ***********  *******:*** :*:::**:.******* ********:****:****

TbACLB1       AIGTLFMDLLSGSGMFSKQEIDEIVNIGYLNGLFVLARSIGLIGHTFDQKRLKQPLYRHP     600
OsACLB1       AIGSLFLDLLSGSGMFSKQEIDEIVEIGYLNGLFVLARSIGLIGHTFDQKRLKQPLYRHP     600
AtACLB2       AIGSLFLDLLAGSGMFTKQEIDEIVQIGYLNGLFVLARSIGLIGHTFDQKRLKQPLYRHP     600
AtACLB1       AIGSLFLDLLAGSGMFTKQEIDEIVQIGYLNGLFVLARSIGLIGHTFDQKRLKQPLYRHP     600
              ***:**:***:*****:********:**********************************

TbACLB1       WEDVLYTK     608
OsACLB1       WEDVLYTK     608
AtACLB2       WEDVLYTK     608
AtACLB1       WEDVLYTK     608
              ********
```

C

```
TbAACT1      MAPAAATNGSDSIKPRDVCIVGVARTPLGGFLGSLSSLPATKLGSIAIESALKRANVDPS        60
AtAACT1      ---MAPPVSDDSLQPRDVCVVGVARTPIGDFLGSLSSLTATRLGSIAIQAALKRAHVDPA        57
AtAACT2      -----MAHTSESVNPRDVCIVGVARTPMGGFLGSLSSLPATKLGSLAIAAALKRANVDPA        55
HbAACT1      ----MAPVAAAEIKPRDVCIVGVARTPMGGFLGLLSTLPATKLGSIAIEAALKRASVDPS        56
                        .::*****:*******:*.*** **:* **:***:** :***** ***:

TbAACT1      LVQEVIFGNVLGANLGQGPARQAALGAGIPNTVVSTTVNKVCASGMKATMLAAQSIQLGI        120
AtAACT1      LVEEVFFGNVLTANLGQAPARQAALGAGIPYSVICTTINKVCAAGMKSVMLASQSIQLGL        117
AtAACT2      LVQEVVFGNVLSANLGQAPARQAALGAGIPNSVICTTVNKVCASGMKAVMIAAQSIQLGI        115
HbAACT1      LVQEVFFGNVLSANLGQAPARQAALGAGIPNSVVCTTVNKVCASGMKATMLAAQSIQLGI        116
             **:**.***** *****.************ :*:.**:*****:***:.*:*:******:

TbAACT1      NDVVVAGGMESMSNVPKYISEARKGSKFGHDTLVDGIMKDGLWDCFNDFKMGSAAEICAD        180
AtAACT1      NDIVVAGGMESMSNVPKYLPDARRGSRLGHDTVVDGMMKDGLWDVYNDFGMGVCGEICAD        177
AtAACT2      NDVVVAGGMESMSNTPKYLAEARKGSRFGHDSLVDGMLKDGLWDVYNDCGMGSCAELCAE        175
HbAACT1      NDVVVAGGMESMSNAPKYLAEARKGSRLGHDSLVDGMLKDGLWDVYNDVGMGSCAEICAD        176
             **:***********.***: :**:**::***::***::****** :**  ** ..*:**:

TbAACT1      TYELTREHQDDYAVQSFERGIAARDSGAFEWEITPVEVPGPRGRPSTIVDKDDDLSKFDP        240
AtAACT1      QYRITREEQDAYAIQSFERGIAAQNTQLFAWEIVPVEVSTGRGRPSVVIDKDEGLGKFDA        237
AtAACT2      KFQITREQQDDYAVQSFERGIAAQEAGAFTWEIVPVEVSGGRGRPSTIVDKDEGLGKFDA        235
HbAACT1      NHSITREDQDKFAIHSFERGIAAQESGAFAWEIVPVEVSGGRGKPLTIVDKDEGLGKFDP        236
              . :***.** :*::********:::  * ***.****   **:* .::***:.*.***

TbAACT1      AKLRKLRPAFKENGGSVTAGNSSGINDGAAALVLVSGEKAIELGLKVIAKVSGYADAEQA        300
AtAACT1      AKLKKLRPSFKEDGGSVTAGNASSISDGAAALVLVSGEKALELGLHVIAKIRGYADAAQA        297
AtAACT2      AKLRKLRPSFKENGGTVTAGNASSISDGAAALVLVSGEKALQLGLLVLAKIKGYGDAAQE        295
HbAACT1      VKLRKLRPSFKENGGTVTAGNASSISDGAAALILVSGETALKLGLQVIAKIRGYADAAQA        296
             .**:****:***:**:*****:*.*.******:*****.*::*** *:**: **.** *

TbAACT1      PELFTTSPALAIPKAISRAGLEASQIDFYEINEAFAAVALANQKLLNLDSAKLNVHGGGV        360
AtAACT1      PELFTTTPALAIPKAIKRAGLDASQVDYYEINEAFSVVALANQKLLGLDPERLNAHGGAV        357
AtAACT2      PEFFTTAPALAIPKAIAHAGLESSQVDYYEINEAFAVVALANQKLLGIAPEKVNVNGGAV        355
HbAACT1      PELFTTAPALAIPKTIANAGLDASQVDYYEINEAFAVVALANQKLLGLNPEKVNVHGGAV        356
             **:***:*******:* .***::**:*:*******:.*********.:   ::*.:**.*

TbAACT1      SLGHPLGCSGARILVTLLGVLKHKGGKYGAAGVCNGGGGASAFVVELV---------- 408
AtAACT1      SLGHPLGCSGARILVTLLGVLRAKKGKYGVASICNGGGGASALVLEFMSEKTIGYSAL 415
AtAACT2      SLGHPLGCSGARILITLLGILKKRNGKYGVGGVCNGGGGASALVLELL---------- 403
HbAACT1      SLGHPLGCSGARILVTLLGVLRKKNAKYGVGGVCNGGGGASALVVELL---------- 404
             **************:****:*: : .***....:*********:*:*::
```
