# Supplementary figures and images for: Isoprenoid biosynthesis in dandelion latex is enhanced by the overexpression of three key enzymes involved in the mevalonate pathway
Source: BMC Plant Biol. 2017 May 22;17:88. doi: 10.1186/s12870-017-1036-0 (PMC5441070; doi:10.1186/s12870-017-1036-0)

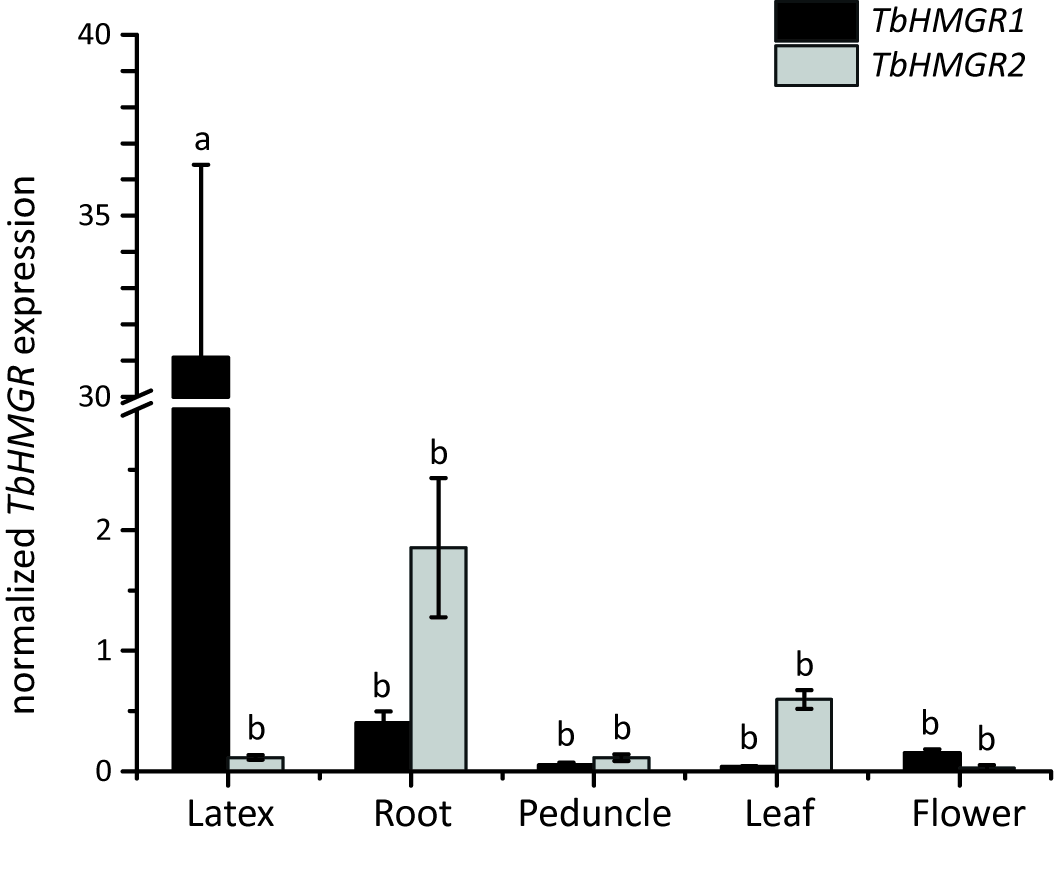

Supplement: Supplementary file 4 — Spatial HMGR expression profile in 10-week-old wild-type T. brevicorniculatum plants. Normalized HMGR1 and HMGR2 mRNA levels in latex, roots, peduncles, leaves and flowers were determined by qRT-PCR. The corresponding mRNA levels were normalized against the constitutive genes elongation factor 1 α (TbEF1α), glyceraldehyde-3-phosphate dehydrogenase (TbGAPDH) and ribosomal protein L27 (TbRP) from T. brevicorniculatum. Bars represent standard errors (n = three biological replicates). Normal distribution at p < 0.05 was assessed using the Kolmogorov-Smirnov test. Different letters indicate a significant difference (ANOVA with Tukey’s honest significant difference test (p < 0.01)). (TIFF 187 kb) [file 12870_2017_1036_MOESM4_ESM.tif]

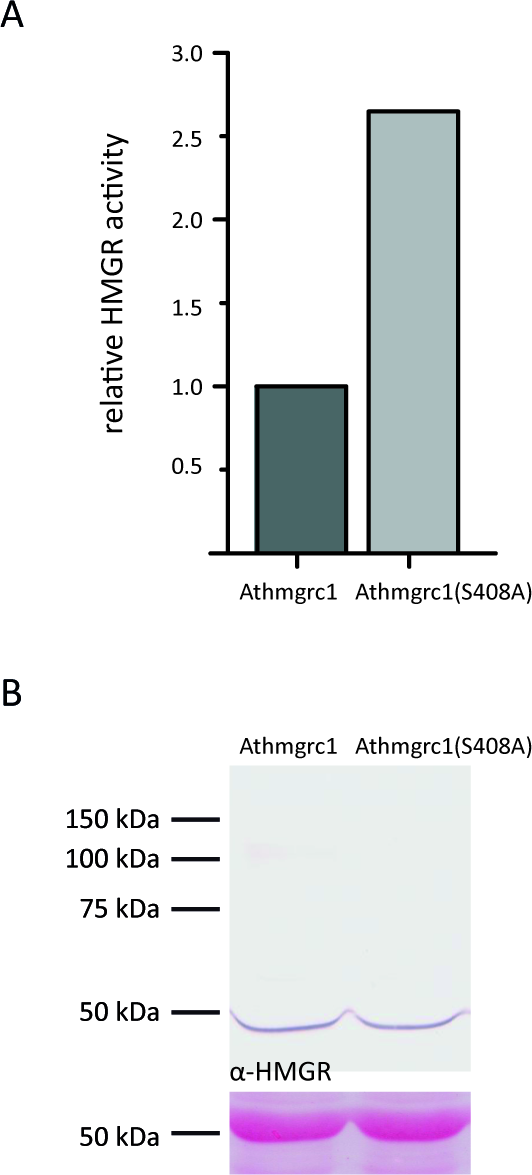

Supplement: Supplementary file 5 — Relative HMGR activity of two different Athmgrc1 variants in the heterologous N. benthamiana system. A: HMGR activity measured in N. benthamiana leaf extracts following the transient expression of Athmgrc1 constructs for 1 week. Athmgrc1, catalytic domain of AtHMGR1; Athmgrc1(S408A), catalytic domain of AtHMGR1 with a serine to alanine substitution at position 408. B: Protein extracts from N. benthamiana leaves transiently expressing Athmgrc1 variants analyzed by SDS-PAGE and western blot (upper part) using an antibody against HMGR. Ponceau S staining after protein transfer is shown below. Numbers on the left refer to molecular weight markers. (TIFF 116 kb) [file 12870_2017_1036_MOESM5_ESM.tif]

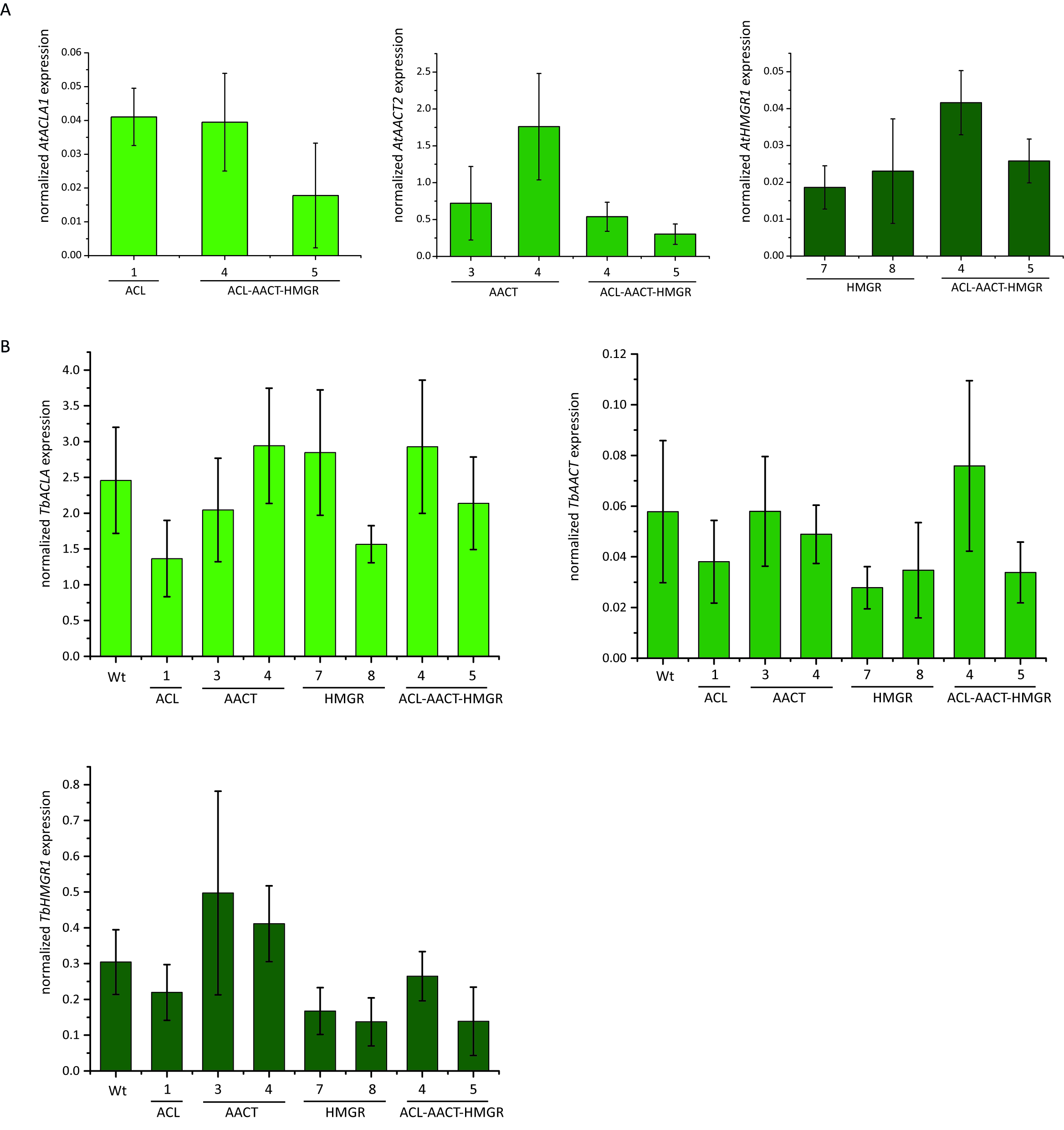

Supplement: Supplementary file 6 — Normalized ACL, AACT and HMGR mRNA levels in transgenic and wild-type T. brevicorniculatum plants quantified by qRT-PCR. The corresponding mRNA levels were normalized against the constitutive gene elongation factor 1 α (TbEF1α) from T. brevicorniculatum. Bars represent standard errors (n = three biological replicates). A: AtACLA1, AtAACT2 and AtHMGR1 transgene mRNA levels in transgenic lines. No significant differences at p < 0.05 were detected among the transgenic lines using the Mann-Whitney U test. B: Endogenous TbACLA, TbAACT and TbHMGR1 mRNA levels in all transgenic lines and wild-type (Wt) plants. No significant differences at p < 0.05 were detected between the wild-type plants and transgenic lines using the Mann-Whitney U test. (TIFF 1510 kb) [file 12870_2017_1036_MOESM6_ESM.tif]

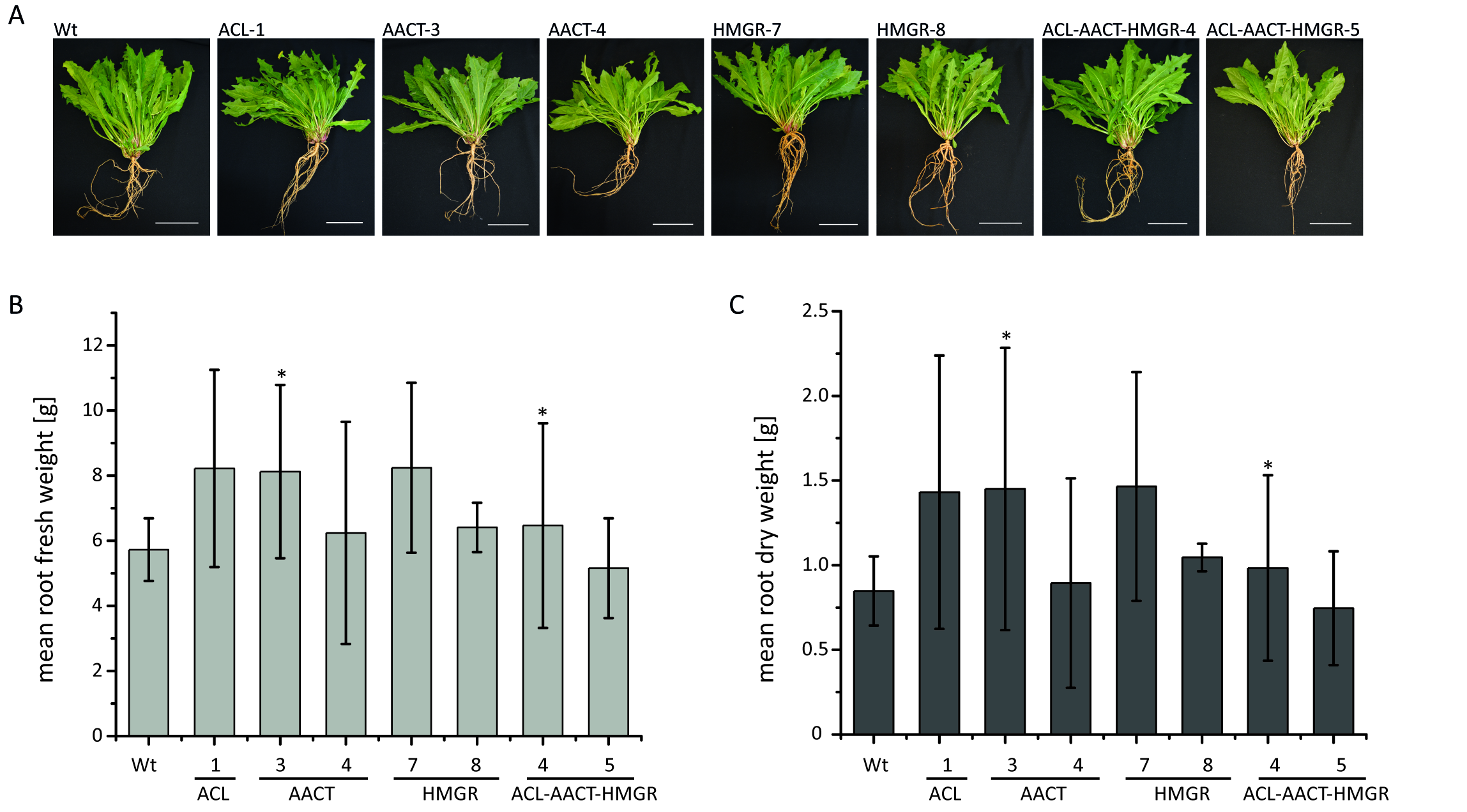

Supplement: Supplementary file 7 — Root morphology and weight of 12-week-old wild-type and transgenic T. brevicorniculatum plants. A: Cleaned roots were harvested and one representative plant from each line was photographed. Scale bar: 10 cm. B: Mean root fresh weight and C: mean root dry weight of harvested roots. Bars represent standard errors (n = three plants; asterisks indicate n = two plants). No significant differences at p < 0.05 were detected between wild-type (Wt) plants and transgenic lines using the Mann-Whitney U test. (TIFF 3459 kb) [file 12870_2017_1036_MOESM7_ESM.tif]

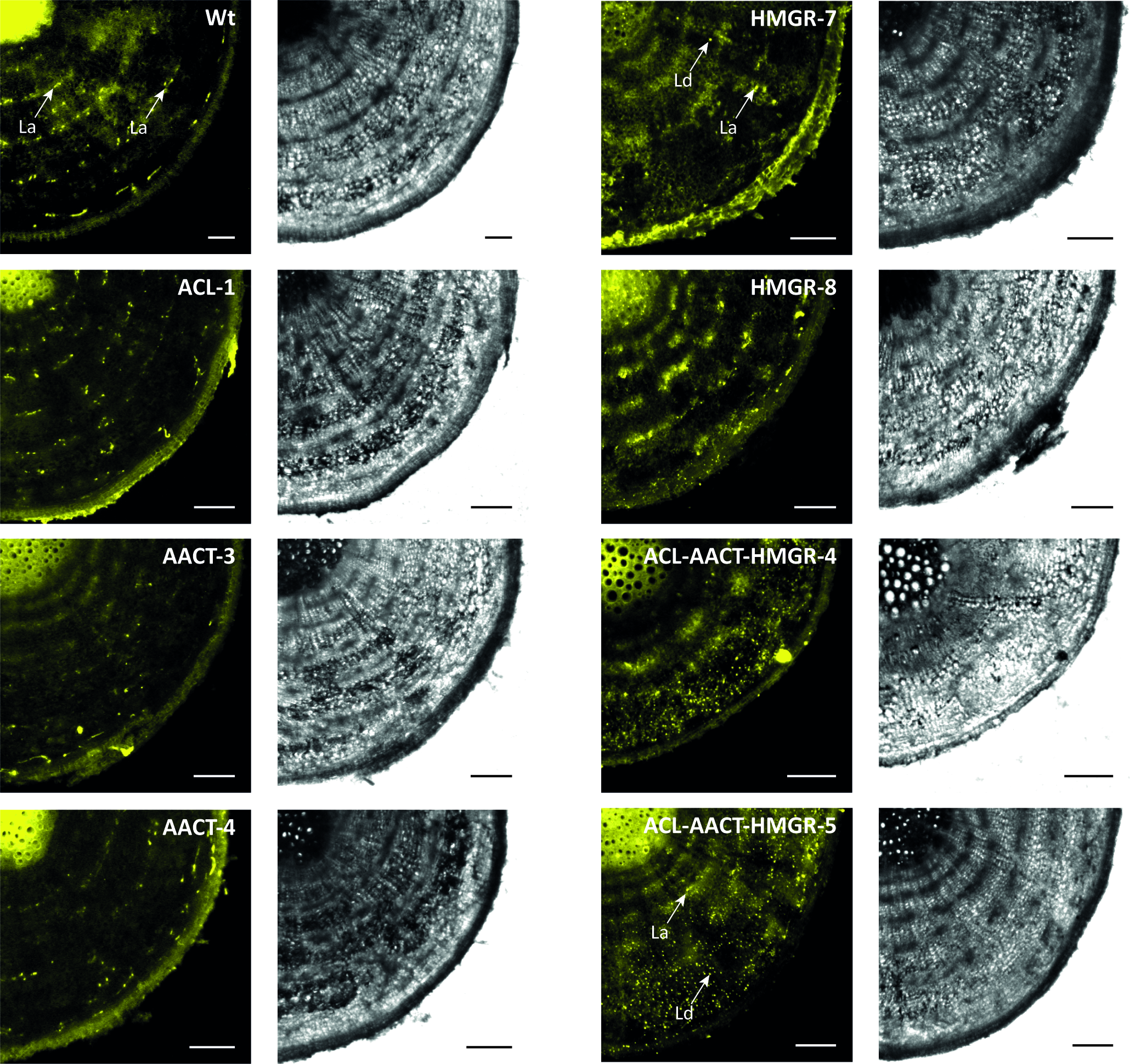

Supplement: Supplementary file 8 — Root cross-sections of 12-week-old wild-type and transgenic T. brevicorniculatum plants. Staining was carried out with Nile red and one representative cross-section is shown for each line. Wt, wild-type; La, laticifer; Ld, lipid droplet. Scale bar: 250 μm.. (TIFF 5280 kb) [file 12870_2017_1036_MOESM8_ESM.tif]
